# Predmoter—cross-species prediction of plant promoter and enhancer regions

**DOI:** 10.1093/bioadv/vbae074

**Published:** 2024-05-24

**Authors:** Felicitas Kindel, Sebastian Triesch, Urte Schlüter, Laura Alexandra Randarevitch, Vanessa Reichel-Deland, Andreas P M Weber, Alisandra K Denton

**Affiliations:** Institute of Plant Biochemistry, Math.-Nat. Faculty, Heinrich Heine University, Düsseldorf 40225, Germany; Institute of Plant Biochemistry, Math.-Nat. Faculty, Heinrich Heine University, Düsseldorf 40225, Germany; Cluster of Excellence on Plant Sciences (CEPLAS), Germany; Institute of Plant Biochemistry, Math.-Nat. Faculty, Heinrich Heine University, Düsseldorf 40225, Germany; Cluster of Excellence on Plant Sciences (CEPLAS), Germany; Institute of Population Genetics, Math.-Nat. Faculty, Heinrich Heine University, Düsseldorf 40225, Germany; Institute of Plant Biochemistry, Math.-Nat. Faculty, Heinrich Heine University, Düsseldorf 40225, Germany; Institute of Plant Biochemistry, Math.-Nat. Faculty, Heinrich Heine University, Düsseldorf 40225, Germany; Cluster of Excellence on Plant Sciences (CEPLAS), Germany; Institute of Plant Biochemistry, Math.-Nat. Faculty, Heinrich Heine University, Düsseldorf 40225, Germany; Cluster of Excellence on Plant Sciences (CEPLAS), Germany; Valence Labs, Montréal, Québec H2S 3H1, Canada

## Abstract

**Motivation:**

Identifying *cis*-regulatory elements (CREs) is crucial for analyzing gene regulatory networks. Next generation sequencing methods were developed to identify CREs but represent a considerable expenditure for targeted analysis of few genomic loci. Thus, predicting the outputs of these methods would significantly cut costs and time investment.

**Results:**

We present Predmoter, a deep neural network that predicts base-wise Assay for Transposase Accessible Chromatin using sequencing (ATAC-seq) and histone Chromatin immunoprecipitation DNA-sequencing (ChIP-seq) read coverage for plant genomes. Predmoter uses only the DNA sequence as input. We trained our final model on 21 species for 13 of which ATAC-seq data and for 17 of which ChIP-seq data was publicly available. We evaluated our models on *Arabidopsis thaliana* and *Oryza sativa*. Our best models showed accurate predictions in peak position and pattern for ATAC- and histone ChIP-seq. Annotating putatively accessible chromatin regions provides valuable input for the identification of CREs. In conjunction with other *in silico* data, this can significantly reduce the search space for experimentally verifiable DNA–protein interaction pairs.

**Availability and implementation:**

The source code for Predmoter is available at: https://github.com/weberlab-hhu/Predmoter. Predmoter takes a fasta file as input and outputs h5, and optionally bigWig and bedGraph files.

## 1 Introduction

Despite large genomic and epigenomic studies being published in all fields of biology, the identification of *cis*-regulatory sequences and their influence on gene regulation is still a major challenge. The discovery of new *cis*-regulatory elements (CREs) can reveal targets for genetic engineering and breeding supporting optimization of plant growth as well as stress and pathogen resistance.

Two important locations of CREs are promoters and enhancers. Promoters are historically defined to serve transcription initiation (Jacob *et al.* 1964, Epstein and Beckwith 1968, Ippen *et al.* 1968). The core promoter is a region of 50 to 100 base pairs (bp) upstream from the transcription start site (TSS) (Dynan and Tjian 1985, Struhl 1995). We refer here to promoter as the assembly of individual transcription factor (TF) binding sites, i.e. CREs, upstream of a gene that entirely or partially drive local transcription initiation. This region contains at least the core promoter. Conversely, enhancers can increase transcription levels from a given promoter. They were found to act in either orientation and at many positions. The first discovered enhancer sequence was found in *Escherichia coli*, and it could act up to 1400 bp upstream or 3300 bp downstream from the TSS (Banerji *et al.* 1981). An example distal enhancer in plants is acting 140 kbp upstream of the *bx1* gene in *Zea mays* (Zheng *et al.* 2015). Whereas the core promoter mostly coordinates expression of the adjacent gene, enhancers can regulate gene expression of multiple genes.

The binary classification of promoters and enhancers has since been challenged. Promoters with high enhancer strengths (Engreitz *et al.* 2016, Dao *et al.* 2017, Diao *et al.* 2017) and active enhancers driving local transcription initiation at their boundaries (Kim *et al.* 2010, Santa *et al.* 2010, Andersson *et al.* 2014) have been reported. Promoters and enhancers usually are both found in accessible chromatin regions (ACRs), where the DNA is accessible to TFs (Gross and Garrard 1988, Cockerill 2011, Song *et al.* 2011). Both promoter and enhancer regions are marked by different histone modifications. Histone H3 trimethylated at lysine 4 (H3K4me3) is primarily present at active genes, while H3K4me2 occurs at both inactive and active euchromatic genes (Santos-Rosa *et al.* 2002). Both can be detected in the core promoter and the coding region of genes. Enhancers are instead marked by H3K4me1 (Heintzman *et al.* 2009). Active enhancers are additionally marked by an acetylation of H3K27 (H3K27ac) (Rada-Iglesias *et al.* 2010). Poised or inactive enhancers are in contrast marked by the absence of H3K27ac, instead showing an enrichment of H3K27 trimethylation (H3K27me3) (Creyghton *et al.* 2010, Rada-Iglesias *et al.* 2010). However, H3K4me1 was found to not commonly be associated with distal ACRs in plants (Lu *et al.* 2019).

Assay for Transposase Accessible Chromatin using sequencing (ATAC-seq) is a common method to identify ACRs genome-wide (Buenrostro *et al.* 2013). It is faster and more sensitive than previous methods like DNase I hypersensitive sites sequencing (DNase-seq) (Crawford *et al.* 2006) or formaldehyde-assisted isolation of regulatory elements (FAIRE-seq) (Giresi *et al.* 2007). ATAC-seq uses hyperactive mutant Tn5-transposase, which cuts the DNA primarily in ACRs and ligates adapters to the cut DNA fragment (Buenrostro *et al.* 2013). The resulting fragments are amplified by PCR creating a sequencing library. In contrast to ATAC-seq, which outputs ACRs, chromatin immunoprecipitation DNA-sequencing (ChIP-seq) (Kim *et al.* 2004, Johnson *et al.* 2007, Robertson *et al.* 2007) is used to investigate how proteins that interact with the DNA regions of interest regulate target gene expression. Proteins attached to the DNA are crosslinked with the DNA, the DNA is sheared, the proteins are immunoprecipitated and unlinked, so the DNA can be amplified and sequenced (Kim *et al.* 2004, Johnson *et al.* 2007, Robertson *et al.* 2007). Depending on the assay, either TF or histone antibodies are used in immunoprecipitation. Promoter as well as enhancer specific histone modifications can be identified using ChIP-seq.

Deep learning (DL) is a part of machine learning using artificial neural networks (NNs) that have multiple hidden layers creating a deep neural network (DNN) architecture (Schulz and Behnke 2012). *In silico* identification of promoter and enhancer sequences using DL was attempted in several studies. Most tools, like DeePromoter (Oubounyt *et al.* 2019), Cr-Prom (Shujaat *et al.* 2021), Depicter (Zhu *et al.* 2021), HPMI (Wang *et al.* 2022), or iProm-Zea (Kim *et al.* 2022), predicted promoters as a sequence stretch around the TSS. The networks in these studies performed a fundamentally different predictive task than actual promoter sequence prediction. Meanwhile, recent enhancer predicting networks, like PREPRINT (Osmala and Lähdesmäki 2020), the cross-species predicting CrepHAN (Hong *et al.* 2021) or iEnhancer-ELM (Li *et al.* 2023), were trained on experimentally verified enhancers. All these studies utilize human and/or other mammalian enhancers. Recent plant enhancer predicting networks, RicENN (Gao *et al.* 2022) and AthEDL (Chen *et al.* 2022), only utilized verified enhancers of *Oryza sativa* or *Arabidopsis thaliana*. Enhancer datasets of a diverse range of plant species are so far not publicly available. Other approaches of predicting regulatory factor binding activity (Hiranuma *et al.* 2017), predicting enhancer regions (Thibodeau *et al.* 2018), predicting single-cell chromatin accessibility (Yuan and Kelley 2022), or predicting transcription-factor binding on a genomic scale (Cazares *et al.* 2023) utilized ATAC-seq data in conjunction with DNA sequence information. However, these networks only utilize ATAC-seq data from human samples. Furthermore, the Enformer DNN can predict gene expression and chromatin states, represented as multiple genomic coverage tracks like H3K27ac coverage, in humans and mice from DNA sequences (Avsec *et al.* 2021). Plant research keeps lagging behind research in mammalian species in this field and a DNN focused on predicting plant CREs would be a first step to alleviate this underrepresentation. Moreover, generating ATAC- and ChIP-seq libraries is costly and time consuming and a DNN predicting plant ATAC- and ChIP-seq read coverage directly from the genomic DNA sequence would circumvent these constraints. To date, no such model has been reported.

Here we present Predmoter, a tool used for cross-species base-wise prediction of plant ATAC- and/or H3K4me3 ChIP-seq read coverage, using the genomic DNA sequence as input. We utilized publicly available ATAC- and ChIP-seq data to infer plant promoter and enhancer regions. We trained our final model on ATAC-seq data from 13 different plant species and ChIP-seq data from 17 plant species.

## 2 Methods

### 2.1 Data

#### 2.1.1 Data overview and preprocessing

The entire dataset consisted of 25 plant genomes, for 17 of which genome-wide ATAC-seq data was publicly available and for 21 of which genome-wide ChIP-seq (H3K4me3) data was publicly available (see Table 1 and Supplementary Table S2). A wide variety of tissues and treatments were used in these ATAC- and ChIP-seq experiments which are listed in Supplementary Table S3.

**Table 1.** Plant genomes and available datasets.

Domain	Species	ATAC-seq	ChIP-seq (H3K4me3)
Algae	*Bigelowiella natans*	**✔**	
	*Chlamydomonas reinhardtii*		**✔**
Mosses	*Marchantia polymorpha*	**✔**	**✔**
Monocots	*Brachypodium distachyon*	**✔**	**✔**
	*Eragrostis nindensis*	**✔**	**✔**
	*Oropetium thomaeum*	**✔**	
	*Oryza brachyantha*		**✔**
	*Oryza sativa*	**✔**	**✔**
	*Panicum miliaceum*	**✔**	
	*Setaria italica*		**✔**
	*Sorghum bicolor*	**✔**	
	*Spirodela polyrhiza*	**✔**	**✔**
	*Zea mays*	**✔**	**✔**
Dicots	*Actinidia chinensis*	**✔**	**✔**
	*Arabidopsis thaliana*	**✔**	**✔**
	*Brassica napus*	**✔**	**✔**
	*Brassica oleracea*		**✔**
	*Brassica rapa*		**✔**
	*Glycine max*	**✔**	**✔**
	*Malus domestica*	**✔**	**✔**
	*Medicago truncatula*	**✔**	**✔**
	*Prunus persica*		**✔**
	*Pyrus x bretschneideri*		**✔**
	*Sesamum indicum*		**✔**
	*Solanum lycopersicum*	**✔**	**✔**

The NGS data was downloaded from the sequence read archive (SRA) using the SRA-Toolkit 3.0.0 (https://github.com/ncbi/sra-tools/wiki/01.-Downloading-SRA-Toolkit). The reads were trimmed with Trimmomatic 0.36 (Bolger *et al.* 2014) and quality controlled using FastQC 0.11.9 (Andrews 2010) and MultiQC (Ewels *et al.* 2016). If the reads passed quality control, they were mapped to the reference genome using BWA 2.1 (Md *et al.* 2019). Conversion to bam files was performed using SamTools 1.6 (Danecek *et al.* 2021). The Picard Toolkit (Broad Institute ed 2019) was used to mark duplicates. The duplicates, unmapped reads, non-primary alignments and reads not passing platform quality checks were removed with SamTools. Plots for quality control were generated using deepTools 3.5.3 (Ramírez *et al.* 2016) and the necessary genome annotations were generated using Helixer v.0.3.1 (Stiehler *et al.* 2021, Holst *et al.* 2023). ATAC-seq data was deemed of high enough quality if the average coverage enrichment ±3 kbp around the TSS showed the expected peak and the average peak read coverage was at least 2.5 times the background coverage. The quality control for ChIP-seq data was performed using the same criteria. A detailed data preprocessing documentation is available at: https://github.com/weberlab-hhu/Predmoter/blob/main/docs/data_preprocessing.md. The plant genome fasta files and final NGS data bam files were converted to h5 files using Helixer (Stiehler *et al.* 2021, Holst *et al.* 2023). The ATAC-seq reads were shifted +4 bp on the positive strand and −5 bp on the negative strand to adjust the read start sites to represent the center of the transposon binding site (Buenrostro *et al.* 2013). A detailed documentation of the h5 file creation and architecture is available at: https://github.com/weberlab-hhu/Predmoter/blob/main/docs/h5_files.md.

The species used in the development of Predmoter are separated into the four domains algae, mosses, monocots, and dicots. The availability and usage of the species dataset for ATAC- or ChIP-seq is indicated by a check mark.

#### 2.1.2 Filtering flagged sequences

**Figure 1.**
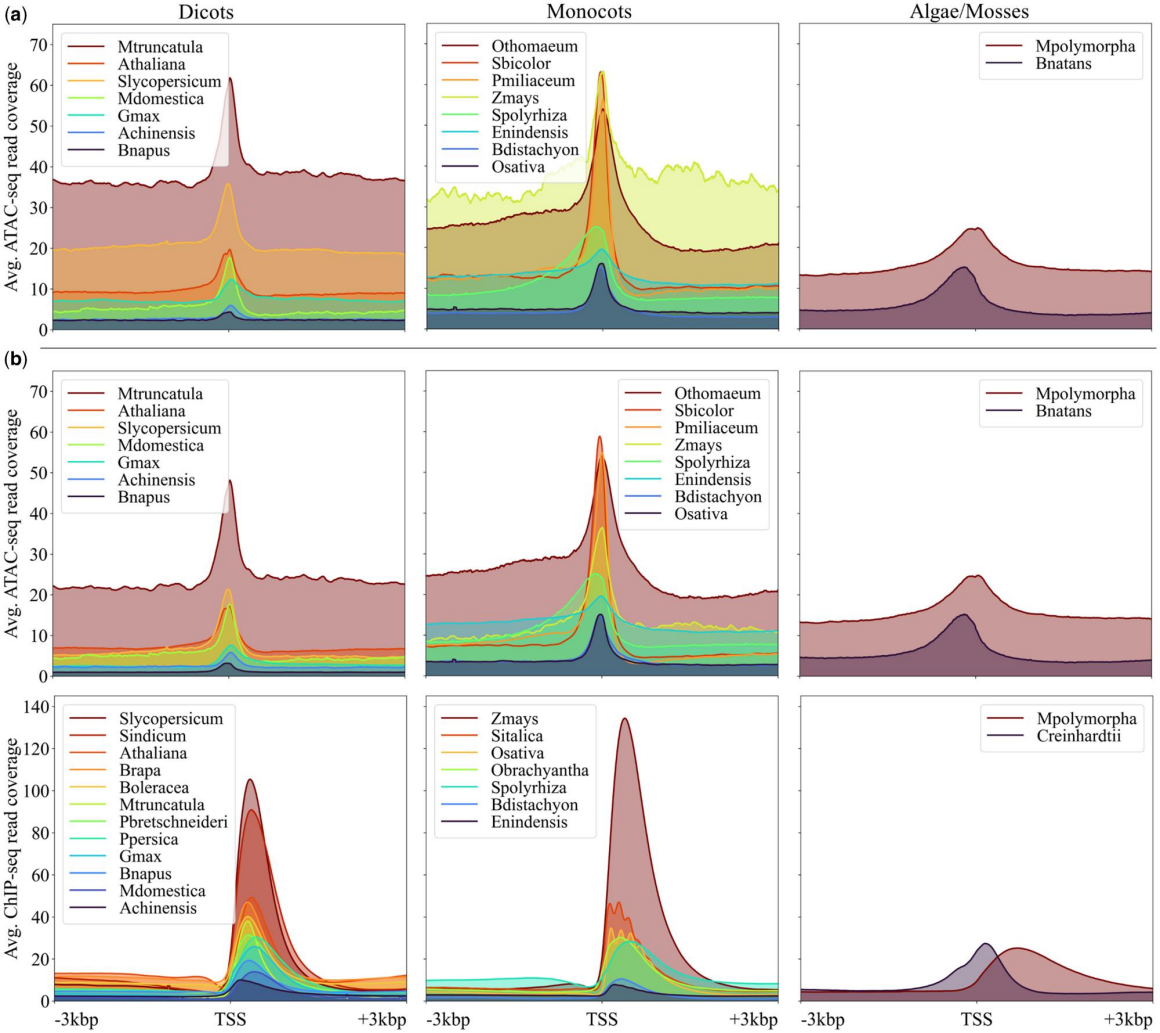
Average ATAC- and ChIP-seq coverage ±3 kpb around the TSS for each species in the dataset. (a) Average ATAC-seq coverage including unplaced scaffolds and non-nuclear sequences. (b) Average ATAC- and ChIP-seq coverage excluding unplaced scaffolds and non-nuclear sequences. The species are sorted into the three categories dicots, monocots, and algae/mosses.

A naïve filtering approach was used to reduce the noise in the dataset. The ATAC-seq data showed high coverage for non-nuclear sequences. The transposase cuts primarily open chromatin (Buenrostro *et al.* 2013) and as such also the chloroplast and mitochondrial genomes. When the organelles were not completely removed before the experiment, the data contained noise in the form of notably higher coverage in these regions. Unplaced scaffolds were also observed to contribute to this noise during the data quality control steps (Fig. 1a).

Therefore, unplaced scaffolds and non-nuclear sequences were flagged during later development stages (see Section 2.2 and Tables 2 and 3). Assemblies on scaffold or contig level, *Bigelowiella natans*, *Eragrostis nindensis*, *Marchantia polymorpha*, *Oropetium thomaeum*, *Pyrus x bretschneiderii*, and *Spirodela polyrhiza*, were not flagged. The flagged sequences were filtered out (Fig. 1b). The information about the assembly accessions of the unplaced scaffolds and non-nuclear sequences was extracted from the sequence report jsonl files available at the NCBI’s RefSeq or GenBank and added to the h5 file (under “data/blacklist”) via add_blacklist.py in “side_scripts.” The flagged sequences reached around 7% of all genome assemblies used not counting assemblies on scaffold or contig level.

**Table 2.** Model architecture and dataset explanation (short).

Model name	Dataset	Architecture
U-Net	ATAC-seq	3 convolutional layers + 3 transposed convolutional layers
Hybrid	ATAC-seq	U-Net + 2 LSTM layers
BiHybrid	ATAC-seq	U-Net + 2 BiLSTM layers
BiHybrid_02	ATAC-seq	U-Net + 2 BiLSTM layers + 6 batch normalization layers
BiHybrid_03.1 (see Fig. 2)	ATAC-seq	U-Net + 2 BiLSTM layers + 6 batch normalization layers + 1 dropout layer (dropout probability of 0.3)
BiHybrid_03.2	ATAC-seq	U-Net + 2 BiLSTM layers + 6 batch normalization layers + 1 dropout layer (dropout probability of 0.5)
BiHybrid_04	ATAC-seq, filtered flagged sequences*	U-Net + 2 BiLSTM layers + 6 batch normalization layers + 1 dropout layer (dropout probability of 0.3)
BiHybrid_05	ChIP-seq (H3K4me3), filtered flagged sequences*	U-Net + 2 BiLSTM layers + 6 batch normalization layers + 1 dropout layer (dropout probability of 0.3)
Combined	ATAC-seq, ChIP-seq (H3K4me3), filtered flagged sequences*	U-Net + 2 BiLSTM layers + 6 batch normalization layers + 1 dropout layer (dropout probability of 0.3)
Combined_02	ATAC-seq, ChIP-seq (H3K4me3), filtered flagged sequences* (+ additional data)	U-Net + 2 BiLSTM layers + 6 batch normalization layers + 1 dropout layer (dropout probability of 0.3)

**Table 3.** Species selection.

Models	U-Net—BiHybrid_04	BiHybrid_05	Combined	Combined_02
Training species	*B.distachyon*	*B.distachyon*	*B.distachyon*	*A.chinensis*
	*B.napus*	*B.napus*	*B.napus*	*B.distachyon*
	*B.natans*	*B.oleracea*	*B.natans*	*B.napus*
	*E.nindensis*	*B.rapa*	*B.oleracea*	*B.natans*
	*G.max*	*C.reinhardtii*	*B.rapa*	*B.oleracea*
	*M.domestica*	*E.nindensis*	*C.reinhardtii*	*B.rapa*
	*M.polymorpha*	*G.max*	*E.nindensis*	*C.reinhardtii*
	*O.thomaeum*	*M.domestica*	*G.max*	*E.nindensis*
	*S.lycopersicum*	*O.brachyantha*	*M.domestica*	*G.max*
	*Z.mays*	*P.bretschneideri*	*M.polymorpha*	*M.domestica*
		*P.persica*	*O.brachyantha*	*M.polymorpha*
		*S.indicum*	*O.thomaeum*	*O.brachyantha*
		*S.italica*	*P.bretschneideri*	*O.thomaeum*
		*S.lycopersicum*	*P.persica*	*P.bretschneideri*
		*Z.mays*	*S.indicum*	*P.miliaceum*
			*S.italica*	*P.persica*
			*S.lycopersicum*	*S.bicolor*
			*Z.mays*	*S.indicum*
				*S.italica*
				*S.lycopersicum*
				*Z.mays*
Validation species	*M.truncatula*	*M.truncatula*	*M.truncatula*	*M.truncatula*
	*S.polyrhiza*	*S.polyrhiza*	*S.polyrhiza*	*S.polyrhiza*
Test species	*A.thaliana*	*A.thaliana*	*A.thaliana*	*A.thaliana*
	*O.sativa*	*O.sativa*	*O.sativa*	*O.sativa*

### 2.2 Architecture and proposed models

The model architectures were implemented using Pytorch Lightning (Falcon 2019) on top of PyTorch (Paszke *et al.* 2019). The model used supervised learning, a method that connects an input to an output based on example input–output pairs (Russell and Norvig 2016).

The input for the model was a genomic DNA sequence. The nucleotides were encoded into four-dimensional vectors (see Supplementary Table S1). The DNA sequence of a given plant species was cut into subsequences of 21 384 bp. This number was large enough to contain typical gene lengths of plants while being divisible by ten of the numbers from one to twenty. An easily divisible subsequence length is a requirement for Predmoter (see Supplementary Section S1.2). As few chromosomes, scaffolds or contigs were divisible by 21 384 bp, sequence ends as well as short sequences were padded with the vector [0., 0., 0., 0.]. Padded base pairs were masked during training. If a subsequence only contained N bases, here referred to as “gap subsequence,” it was filtered out. Both strands, plus and minus, were used. Since the ATAC- and ChIP-seq data was PCR amplified and as such it was not possible to determine from which strand a read originated, the coverage information was always added to both strands. The model’s predictions for either ATAC-seq, ChIP-seq or both were compared to the experimental read coverage. The target data were represented per sample of experimental data. These were averaged beforehand, resulting in one coverage track per NGS dataset and plant species.

**Figure 2.**
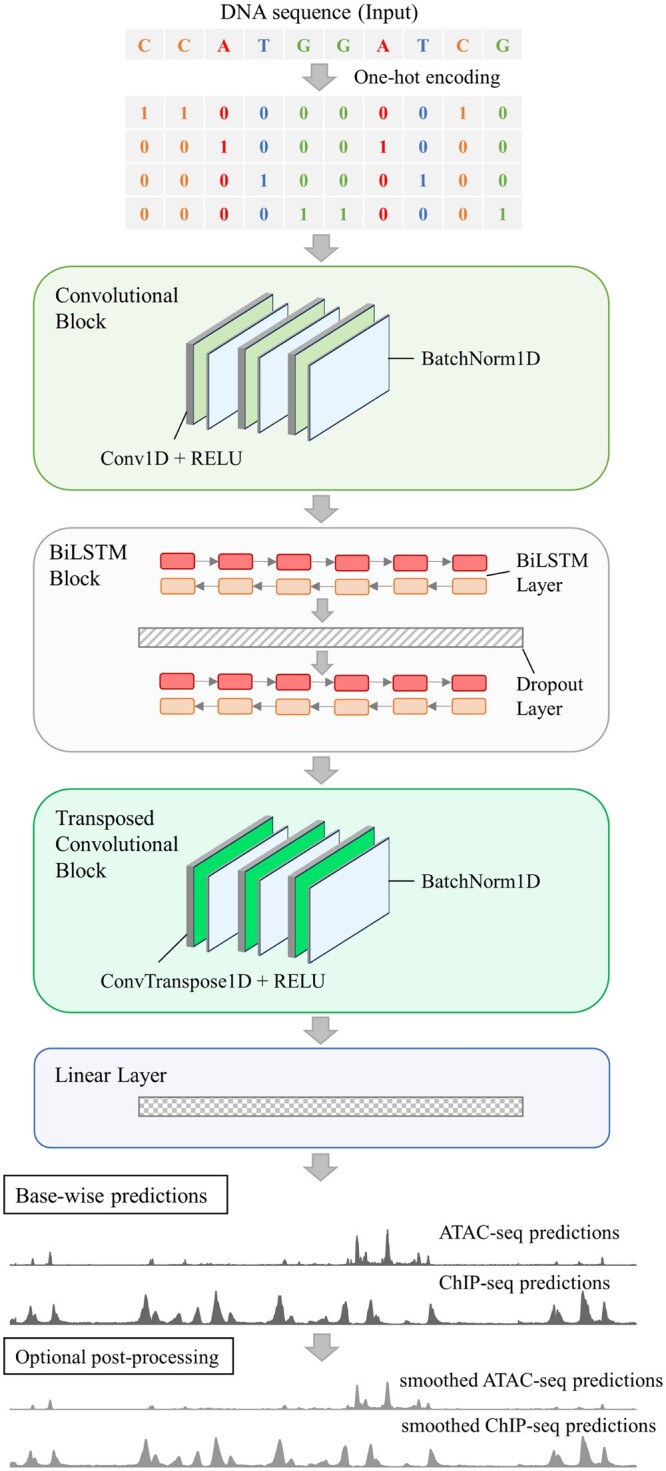
Predmoter architecture and prediction process. The bi-hybrid architecture with batch normalization and dropout is schematically depicted. Not to scale. Hyperparameters are examples and can vary. The base-wise predictions and smoothed predictions are from an example subsequence from *A. thaliana*.

Three main model architectures were examined on their performance. The first architecture consisted of convolutional layers followed by transposed convolutional layers for deconvolution (LeCun *et al.* 1989, LeCun and Bengio 1995). The deconvolution was added to output base-wise predictions. We refer here to this architecture as U-Net. To ensure that the new sequence lengths resulting from a convolution or deconvolution was correct, custom padding formulas were used (Supplementary Section S1.2). Our second approach was a hybrid network. A block of long short-term memory layers (LSTM) (Hochreiter and Schmidhuber 1997) was placed in between a convolutional layer block and a transposed convolutional layer block. The final approach was called bi-hybrid. Its architecture matched the hybrid architecture, except that the LSTM layers were replaced with bidirectional LSTM layers (BiLSTM) (Hochreiter and Schmidhuber 1997, Schuster and Paliwal 1997). Each convolutional and transposed convolutional layer was followed in all three approaches by the ReLU activation function (Glorot *et al.* 2011). Additional augmentations to the bi-hybrid network included adding batch normalization after each convolutional and transposed convolutional layer and adding a dropout layer after each BiLSTM layer except the last (Fig. 2). The Adam algorithm was used as an optimization method (Kingma and Ba 2014). The network’s base-wise predictions can be smoothed via a postprocessing step utilizing a rolling mean of a given window size.

We examined 10 different model setups (Table 2). The best model of each architecture and dataset combination was used to develop the next combination test. The model reaching the highest Pearson’s correlation for the validation set was deemed the best model. Pre-tests showed that including gap subsequences, subsequences of 21 384 bp only containing Ns, led to a considerably lower Pearson’s correlation. The proportion of gap subsequences in the total data was 0.6%. Normalizing the NGS coverage data through a general approach of subtracting the average coverage from the dataset and using a ReLU transformation (Glorot *et al.* 2011) showed notably worse results during previous attempts. The approach of normalizing via an input sample was not feasible due to the considerable lack of available ATAC-seq input samples accompanying the experiments. Therefore, the target data was not adjusted towards its sequencing depth. For more information about the training process see Supplementary Section S1.3.

All models excluded gap subsequences, subsequences of 21 384 bp only containing Ns. For more details on species selection and exact model parameters see Supplementary Table S4. Models excluding subsequences of unplaced scaffolds and non-nuclear sequences during training and testing are denoted with *.

### 2.3 Species selection

#### 2.3.1 Cross-species prediction models

Ensuring a diverse range of species in the training set, while simultaneously reserving enough data for validation and testing to effectively evaluate the models’ generalization ability, proved difficult. At the start of development, the amount of high-quality, publicly available ATAC-seq data was low. Around 60% of the plant ATAC-seq data on SRA available up until July 2023 needed to be discarded after the final quality control. This left the ATAC-seq data of the 14 plant species used in this study. In later development stages 3 more ATAC-seq datasets, from *Actinidia chinensis*, *Panicum miliaceum* and *Sorghum bicolor*, and 2 more ChIP-seq datasets corresponding to acquired ATAC-seq datasets, from *A.chinensis* and *M.polymorpha*, became available. The low availability of high-quality data, especially in early development stages, turned out to be a major hindrance in providing the network with an appropriate amount of data to train on. Data of two species, *A.thaliana* and *O.sativa*, was set aside as a hold-out test set. In doing so, both a dicot and a monocot species with available ATAC- and ChIP-seq datasets could be used for final evaluation. The same applied to the two validation species, the dicot *Medicago truncatula* and the monocot *S.polyrhiza* (Table 3).

**Figure 3.**
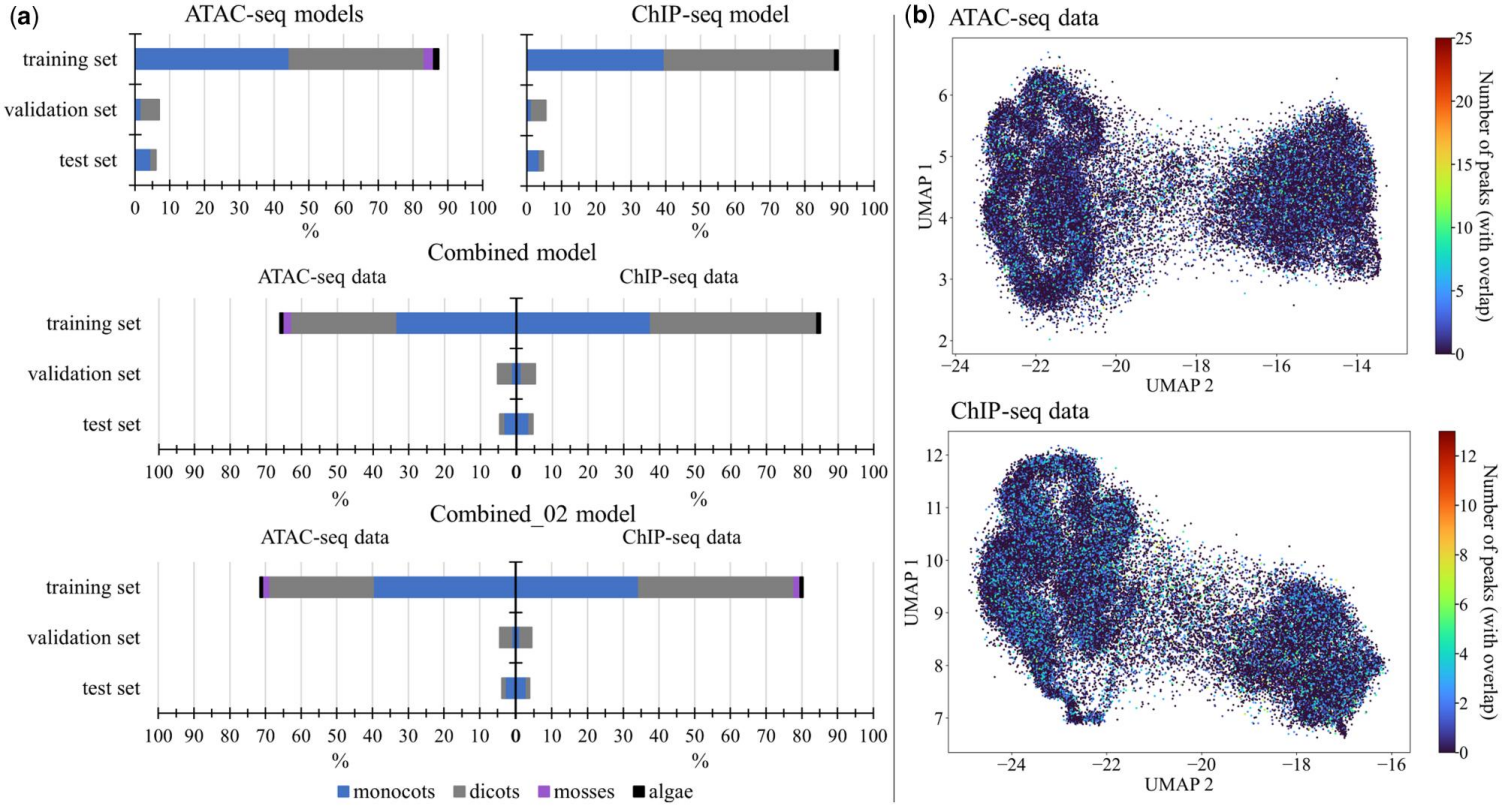
Dataset statistics and visualization. (a) The training, validation, and test split percentages for the ATAC-seq only models, the ChIP-seq only model (BiHybrid_05), the Combined model and the Combined_02 model are subdivided into the four domains monocots, dicots, mosses, and algae. For the Combined models, i.e. the multilabel prediction models, the percentages are shown per dataset, since both datasets were not available for all species (see Table 1). (b) UMAPs of training data. The species for which ATAC- and ChIP-seq data was available were used to create the UMAPs. One point represents one entire one-hot encoded subsequence of 21 384 bp of the training data. The points were colored by the number of peaks present in each subsequence (see Section 2.1.3). Peaks just partially overlapping a subsequence were counted as well.

The resulting training, validation, and test split for the ATAC-seq models, ChIP-seq models and Combined models was around 90% training set, 5% validation set and 5% test set (Fig. 3a).

The model training pairs were visualized using the Uniform Manifold Approximation and Projection (UMAP) learning technique for dimension reduction (McInnes *et al.* 2018). Random training pairs, 5% of each species in the training set, were used to calculate the UMAPS. Gap subsequences and flagged sequences were not included. The chosen parameters were 10 neighbors, 0.1 minimum distance and the Euclidean distance metric. The additional species datasets, added in later development stages, were included. None of the available settings and metrics for UMAP computation showed distinct clusters based on the number of peaks within the input (Fig. 3b).

For the first seven models only the species for which experimental ATAC-seq data of high quality was available up until July of 2023 were trained on. The same applied to the BiHybrid_05 model using ChIP-seq data. The Combined model used both datasets. The Combined_02 model used additional data of four species. Gap subsequences were masked for all models; unplaced scaffolds and non-nuclear sequences were masked starting with model BiHybrid_04.

#### 2.3.2 Intra-species models and leave-one-out cross-validation

Cross-species validation instead of an in-species split for the validation and training data was deemed closer to the real-world use case of predicting ATAC- and ChIP-seq data for an entire species. However, two models were trained using an intra-species training and validation split. These models, IS_10 and IS_20, used 10% and 20% of each species dataset as the validation set respectively. The input files were split using Predmoter's intra_species_train_val_split.py script in “side_scripts.” This method ensured that each sequence ID from the original fasta file was fully assigned to either training or validation set. Since the focus of this study is on cross-species prediction, all 25 plant species were used in leave-one-out cross-validation (LOOCV) to evaluate the best model setup on different species. All these setups were trained on ATAC- and ChIP-seq datasets simultaneously (Table 4). When performing LOOCV the model performance was evaluated on all datasets available in the left-out species.

**Table 4.** Model architecture and dataset explanation (additional models).

Model name	Dataset	Architecture	Comment
IS_10	ATAC-seq, ChIP-seq (H3K4me3), filtered flagged sequences* (+ additional data)	U-Net + 2 BiLSTM layers + 6 batch normalization layers + 1 dropout layer (dropout probability of 0.3)	Intra-species training and validation split (validation set: 10%)
IS_20	ATAC-seq, ChIP-seq (H3K4me3), filtered flagged sequences* (+ additional data)	U-Net + 2 BiLSTM layers + 6 batch normalization layers + 1 dropout layer (dropout probability of 0.3)	Intra-species training and validation split (validation set: 20%)
“25 LOOCV models”	ATAC-seq, ChIP-seq (H3K4me3), filtered flagged sequences* (+ additional data)	U-Net + 2 BiLSTM layers + 6 batch normalization layers + 1 dropout layer (dropout probability of 0.3)	Validation set: each entire species dataset once

All models excluded gap subsequences, subsequences of 21 384 bp only containing Ns, and flagged subsequences. For more details on exact model parameters see Supplementary Section S1.3 and Supplementary Table S4.

### 2.4 Peak calling

Peak calling on predictions and the experimental data was performed with MACS3 (Zhang *et al.* 2008). The sample bam files of the experimental data per species and dataset were merged. Then peaks were called on the merged bam files with MACS3’s “callpeak” command. The parameters for calling ATAC-seq peaks were the BAMPE format, a q-value of 0.01, keeping all duplicates, using the background lambda as local lambda (“no-lambda”) and the ungapped genome size of the species’ genome assembly (see Supplementary Table S2) as mappable genome size. For ChIP-seq peak calling two parameters, broad and a broad cutoff of 0.1, were added. The chosen *q*-value was the default 0.05. The ChIP-seq peaks of the species *S.polyrhiza* and *Chlamydomonas reinhardtii* were called using the format BAM instead of BAMPE. MACS3’s “bdgpeakcall” was used to call peaks on the test species predictions in bedGraph file format. The parameters for peak calling were the same MACS3’s “callpeak” determined for the experimental data, i.e. for paired end reads the minimum length and maximum gap are set to the predicted fragment size (Table 5). The cutoff value, threshold of the minimum read coverage to call a peak, was estimated by plotting the average read coverage of predictions around the TSS (see Fig. 5b).

**Table 5.** Peak calling parameters.

Test species	Dataset	Minimum length	Maximum gap	Cutoff
*Arabidopsis thaliana*	ATAC-seq	149	149	5
	ChIP-seq (H3K4me3)	201	201	15
*Oryza sativa*	ATAC-seq	73	73	15
	ChIP-seq (H3K4me3)	142	142	10

Different cutoff values were also examined. For the ATAC-seq predictions of *A. thaliana*, cutoffs in the range of 1 to 25 with a step of 1 and for *O. sativa* cutoffs in the range of 5 to 200 with a step of 5 and including a cutoff of 1 at the start were chosen. For the ChIP-seq predictions of both species, cutoffs in the range of 5 to 100 with a step of 5 and including a cutoff of 1 at the start were chosen.

The selected parameters of MACS3’s “bdgpeakcall” for each test species and dataset are listed.

### 2.5 Metrics

Five metrics were used to evaluate model performance, the Poisson loss, the Pearson correlation coefficient (Pearson’s *r*), precision, recall, and *F*_1_.

The most prominent peak caller for ChIP-seq data, MACS (Zhang *et al.* 2008), which was also frequently used for ATAC-seq data (Hiranuma *et al.* 2017, Thibodeau *et al.* 2018, Hentges *et al.* 2022), assumes that the ChIP-seq coverage data is Poisson distributed. Therefore, PyTorch’s Poisson negative log likelihood loss function (Poisson loss) was used as the loss function for all models (Equation 1).

(1) $$\mathrm{loss}=\frac{1}{n}\sum_{i=1}^{n}e^{x_{i}}-y_{i}*x_{i}$$

The individual samples of the predictions (x) and the targets (y) are indexed with i. The sample size is denoted with n (https://pytorch.org/docs/stable/generated/torch.nn.PoissonNLLLoss.html). This version of the Poisson loss caused the network to output logarithmic predictions. The desired, actual predictions were thus the exponential of the network’s output. The exponential distribution only consists of positive real numbers like the ATAC- and ChIP-seq read coverage.

To measure the “accuracy” of the model’s predictions, i.e. translating the Poisson loss into a human-readable number, the Pearson’s *r* was chosen (Equation 2), measuring the linear correlation between two variables.

(2) $$r=\frac{\sum_{i=1}^{n}\left(x_{i}-\bar{x}\right)\left(y_{i}-\bar{y}\right)}{\sqrt{\sum_{i=1}^{n}{\left(x_{i}-\bar{x}\right)}^{2}\sum_{i=1}^{n}{\left(y_{i}-\bar{y}\right)}^{2}}+ɛ}$$

The sample size is denoted with n, the individual samples of the predictions (x) and the targets (y) are indexed with i. The additional epsilon (ɛ) equals 1e-8 and is used to avoid a division by zero. A value of 1 represents a perfect positive linear relationship, so Predmoter’s predictions and the experimental NGS coverage data would be identical. A value of 0 means no linear relationship between the predictions and targets. Finally, a value of −1 represents a perfect negative linear relationship.

Precision, recall, and *F*_1_ were used to compare predicted peaks to experimental peaks for both test species (Equations 3–5). A *F*_1_ score of 1 indicates that the predicted peaks are at the same position as the experimental peaks. The lowest score possible is 0. Precision, recall, and *F*_1_ were calculated base-wise. Called peaks were denoted with 1, all other base pairs with 0. A confusion matrix containing the sum of True Positives (TP), False Positives (FP), and False Negatives (FN) for the two classes, peak and no peak, was computed for the average predicted coverage of both strands. Precision and recall were also utilized to plot precision-recall curves (PRC). The area under the precision-recall curve (AUPRC) was calculated using scikit-learn (Pedregosa *et al.* 2011). Flagged sequences were excluded from the calculations (see Section 2.1.2). The baseline AUPRC is equal to the fraction of positives, i.e. the percentage of peaks in the training set (Saito and Rehmsmeier 2015). The peak percentages were calculated using the Predmoter’s compute_peak_f1.py script in “side_scripts.” The percentages are listed in Supplementary Table S8.

(3) $$\mathrm{precision}=\frac{\mathrm{TP}}{\mathrm{TP}+\mathrm{FP}}$$

(4) $$\mathrm{recall}=\frac{\mathrm{TP}}{\mathrm{TP}+\mathrm{FN}}$$

(5) $$F_{1}=2*\frac{\mathrm{precision}*\mathrm{recall}}{\mathrm{precision}+\mathrm{recall}}$$

## 3 Results

**Figure 4.**
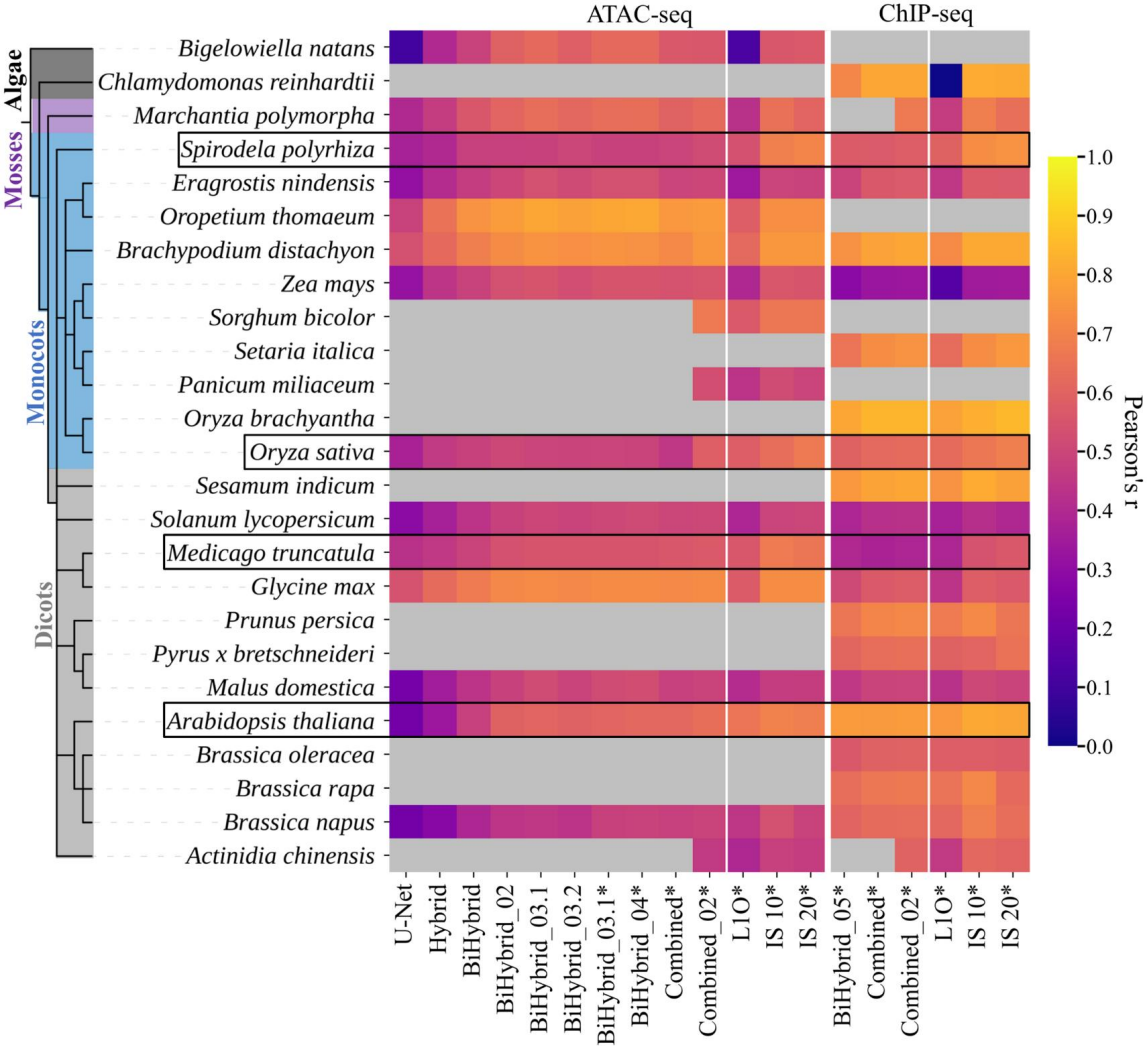
Performance of the best models per model setup across all species. The performance is measured via the Pearson correlation coefficient by comparing the experimental data (target) with the model’s prediction. Gap subsequences were excluded during testing. Results marked with * also excluded flagged subsequences (see Section 2.1.2). The validation and test species metrics are bordered by black boxes. The left block shows the results for ATAC-seq, the right one for ChIP-seq (H3K4me3). The performance of the 25 leave-one-out cross validation models (L1O) and two intra-species models (IS_10 and IS_20) is shown in the right section of each block. Grey boxes are used when there was no available high-quality experimental data for the given NGS dataset and species to compare predictions to. The model setups are listed in Table 2; the species selection in Table 3. Tabular results are listed in Supplementary Tables S6 and S7.

Ten different cross-species prediction models were trained and evaluated (see Table 2). A comparison of the first three setups showed that the best base architecture was the BiLSTM layers placed in between a block of convolutional layers and a block of transposed convolutional layers, called “bi-hybrid” in Predmoter (Fig. 4). The architecture used three convolutional, three transposed convolutional and two BiLSTM layers. This setup outperformed the U-Net architecture, which was missing the LSTM layers in the middle, as well as the hybrid architecture that utilized two one-directional LSTM layers. The U-Net performed worst out of all examined models. The model setup BiHybrid_02 added batch normalization after each convolutional and transposed convolutional layer. These additional six layers improved the results further. Introducing a dropout layer with a dropout probability of 30% between the two BiLSTM layers, model architecture BiHybrid_03.1, showed modest improvements. In contrast, the architecture BiHybrid_03.2 with a dropout probability of 50% did not improve the model. Filtering flagged sequences, meaning unplaced scaffolds and non-nuclear sequences, i.e. mitochondrial and chloroplast DNA, in the assembly where possible, was introduced for model ByHybrid_04. Filtering improved the test metrics slightly compared to BiHybrid_03.1. For this comparison the flagged sequences were also once excluded during testing, but not training of BiHybrid_03.1. This final stage of the models’ architecture and development was then used to train on ChIP-seq (H3K4me3) data instead of ATAC-seq data, denoted as model BiHybrid_05. Two Combined models were trained using the setup of BiHybrid_04 and BiHybrid_05, but training on ATAC- and ChIP-seq data simultaneously. For the ChIP-seq data, noise originating from non-nuclear sequences and unplaced scaffolds was not observed. The flagged data, therefore, would have been for the most part another set of the “negative” data with no associated ChIP-seq peaks. As the ATAC- and ChIP-seq data cannot be filtered independently in Predmoter's implementation, filtering of flagged sequences was used for both the BiHybrid_05 and the Combined model to ensure comparability. The Combined model performed better on the ChIP-seq data than the ChIP-seq model BiHybrid_05, but worse for the ATAC-seq data than the previous best ATAC-seq model BiHybrid_04. The Combined_02 model, containing 3 more ATAC-sed datasets and 2 more ChIP-seq datasets in the training set, outperformed all other models.

The results were stable for the validation and test species during leave-one-out cross validation (Fig. 4). The two models using the alga species *B.natans* and *C.reinhardtii* as validation set respectively reached the lowest Pearson’s *r* values of 0.1247 and −0.0379. Intra-species predictions are an easier task as the network does not need to generalize to the same degree neither across biological effects between species nor technical effects like sequencing depth; as expected, intra-species values were between 0.1 and 0.18 higher (Fig. 4). The intra-species model IS_10 trained on 90% of the data from each species and was validated on 10%. It achieved higher validation Pearson’s *r* values than the IS_20 model, which trained on 80% of the data from each species, did for its validation set.

**Figure 5.**
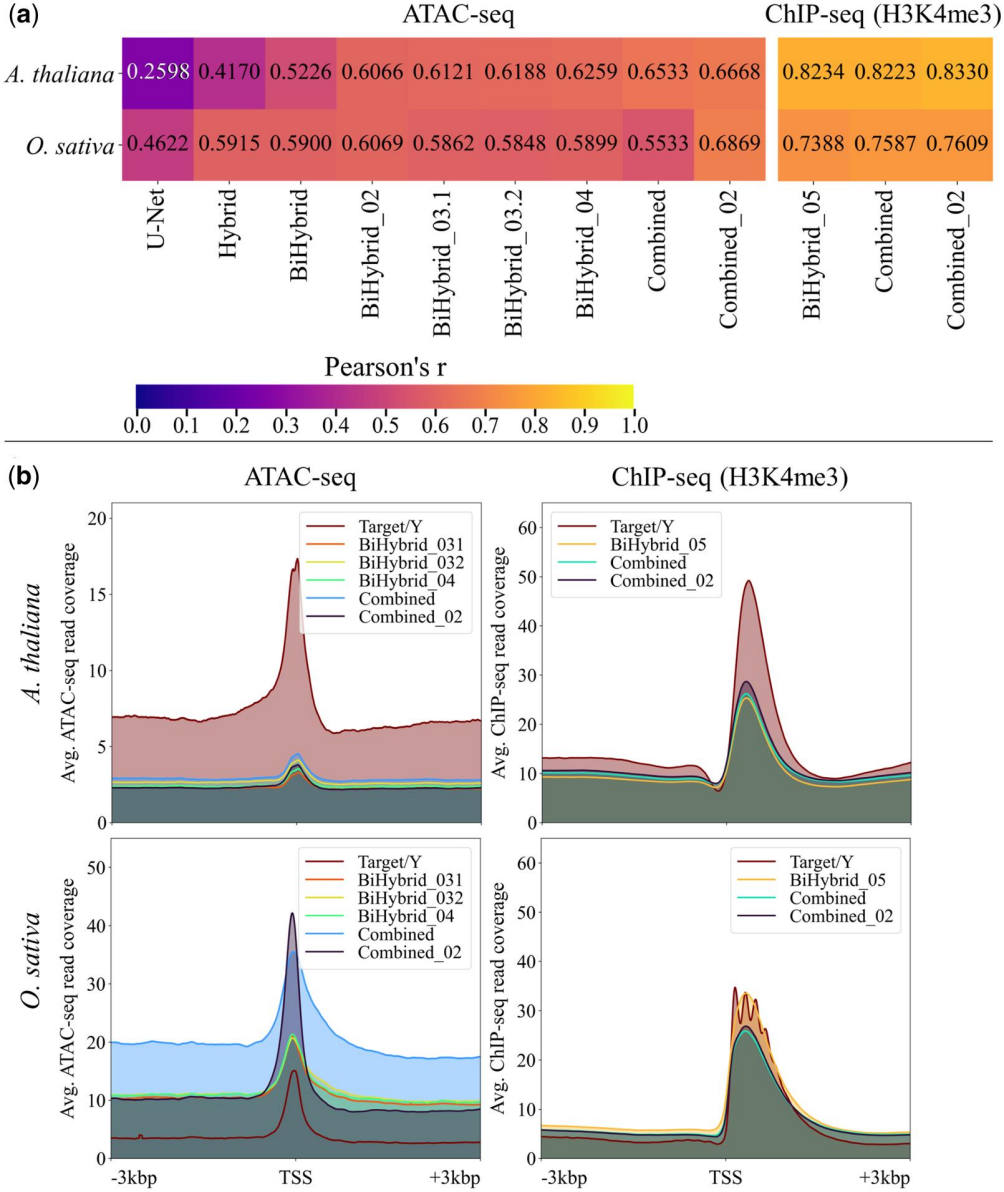
Performance of the best models per model setup and test species +/- 3 kbp around all TSS. (a) The average predicted read coverage for each model and dataset of both strands was compared to the average experimental read coverage of both strands via Pearson’s correlation. (b) The average experimental read coverage (target/year) and predicted ATAC- and ChIP-seq read coverage in reads per bp are shown for *A.thaliana* and *O.sativa*. The predictions of five of the nine best ATAC-seq models and of all three best ChIP-seq models are depicted. See Supplementary Figure S1 for a version of this figure including the predictions of all models. Flagged sequences were excluded from the calculations (see Section 2.1.2).

Next, the predictions for *A.thaliana* and *O.sativa* ±3 kbp around all TSS were examined. The results were stable, when focusing on these regions (Fig. 5a). The Combined_02 model still showed the highest Pearson correlation coefficients, between 0.67 and 0.69 for the ATAC-seq predictions and between 0.76 and 0.83 for the ChIP-seq predictions.

Average coverage enrichment ±3 kbp around the TSS of the ATAC- and ChIP-seq predictions and experimental data of both strands from *A.thaliana* and *O.sativa* showed that the predicted peaks had the similar pattern and were at the same location as the ones from the experimental data (Fig. 5b). For all five depicted ATAC-seq models and all three depicted ChIP-seq models the average read coverage of *A.thaliana* was predicted to be lower than the experimental coverage. The predicted ATAC-seq read coverage of *O.sativa* was higher than the experimental coverage. This applied to all five ATAC-seq models. The amplitudes of the predicted *O.sativa* ChIP-seq read coverage of all three models were close to the experimental read coverage.

**Figure 6.**
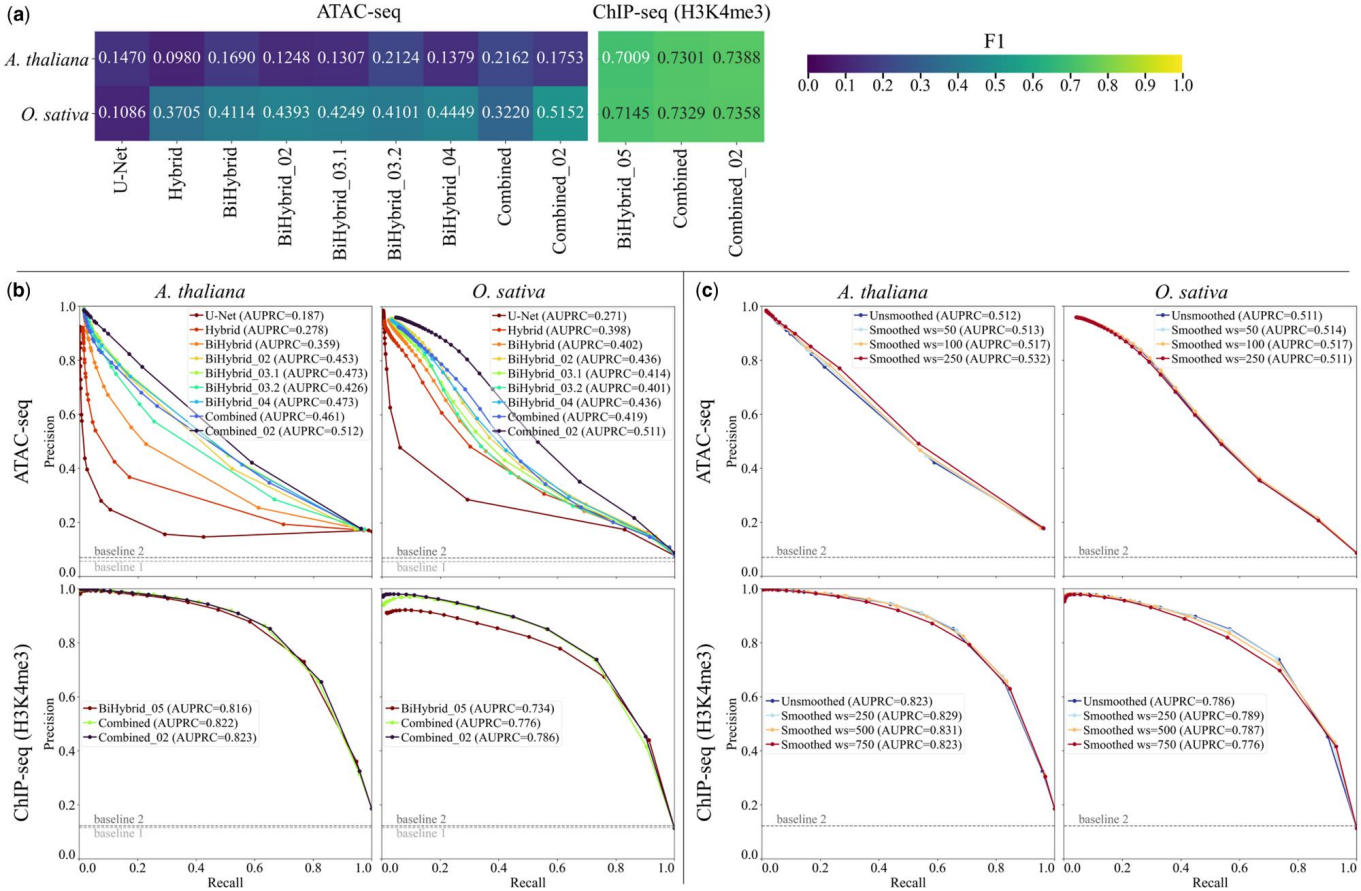
Peak *F*_1_ statistics and precision-recall curves. (a) The *F*_1_ of the predicted peaks versus the experimental peaks was calculated per model, test species and NGS dataset. (b) The precision-recall curves were calculated per test species, model, and dataset. The threshold/cutoff points are marked by circles. The exact parameters and cutoffs used are listed in Section 2.4. Two AUPRC baselines (dashed lines) are depicted. Baseline 2 only applied to the Combined_02 model’s predictions, as this model trained on additional data (see Section 2.3.1). (c) The Combined_02 model’s predictions were smoothed utilizing a rolling mean with a given window size (ws). The precision-recall curves were calculated per test species, window size, and dataset. The threshold/cutoff points are marked by circles. The exact parameters and cutoffs used are listed in Section 2.4. The AUPRC baseline (dashed line) is depicted. Flagged sequences were excluded from peak calling and *F*_1_, precision and recall calculations (see Section 2.1.2).

A base-wise *F*_1_ was calculated to quantify predicted peaks matching experimental peaks (Fig. 6a). The highest *F*_1_ score for the ATAC-seq peaks of *A.thaliana* was the Combined model’s score of 0.2162. For the ATAC-seq peaks of *O.sativa* the Combined_02 model’s predictions resulted in the highest *F*_1_ score of 0.5152. In the case of *A.thaliana*, precision, the rate of false negatives, was notably higher than recall. This applied to all tested models. Precision was also slightly higher than recall for the ChIP-seq predictions for *A.thaliana*. For the ATAC-seq predictions of *O.sativa* recall was higher than precision. Precision and recall were balanced for the ChIP-seq predictions of *O.sativa*. The predicted ChIP-seq peaks showed higher *F*_1_ scores for both test species than the predicted ATAC-seq peaks. The Combined_02 model’s *F*_1_ scores were the highest of all ChIP-seq coverage predicting models.

To understand whether the variation in precision and recall was reflecting fundamental differences in the model performance or simply differences in magnitudes and thresholding of the resulting peaks, we approximated one precision-recall curve per model by shifting the threshold, i.e. the cutoff value of MACS3’s “bdgpeakcall” (see Section 2.4), during peak calling (Fig. 6b). The highest resulting area under the precision-recall curve (AUPRC) of all ATAC-seq models had a value of 0.512 and 0.511 for the best *A.thaliana* and *O.sativa* predictions, respectively; indicating fundamentally similar discriminative performance between species, and that the precision and recall imbalances are addressable by adjusting threshold parameters. The Combined_02 model showed not only the highest AUPRC values for the ATAC-seq predictions for both test species, but also for the H3K4me3 ChIP-seq predictions with values of 0.823 and 0.786. All models achieved higher AUPRC values than the baselines, i.e. the fraction of peaks in the training set (see Section 2.5 and Supplementary Table S9).

To further improve the prediction quality, we implemented a postprocessing step; a rolling mean transformation with a given window size, to smooth the predictions. We tested three different window sizes per NGS dataset; window sizes 50, 100, and 250 for the ATAC-seq predictions and window sizes 250, 500, and 750 for the histone ChIP-seq predictions, as the histone ChIP-seq peaks were broader than ATAC-seq peaks (Fig. 6c). Smoothing the predictions resulted in higher AUPRC values for the ATAC-seq predictions for *A.thaliana*. The ATAC-seq predictions of *O.sativa* improved for window sizes 50 and 100, but not for 250. The ChIP-seq predictions improved for both test species for window sizes 250 and 500, but not for 750.

**Figure 7.**
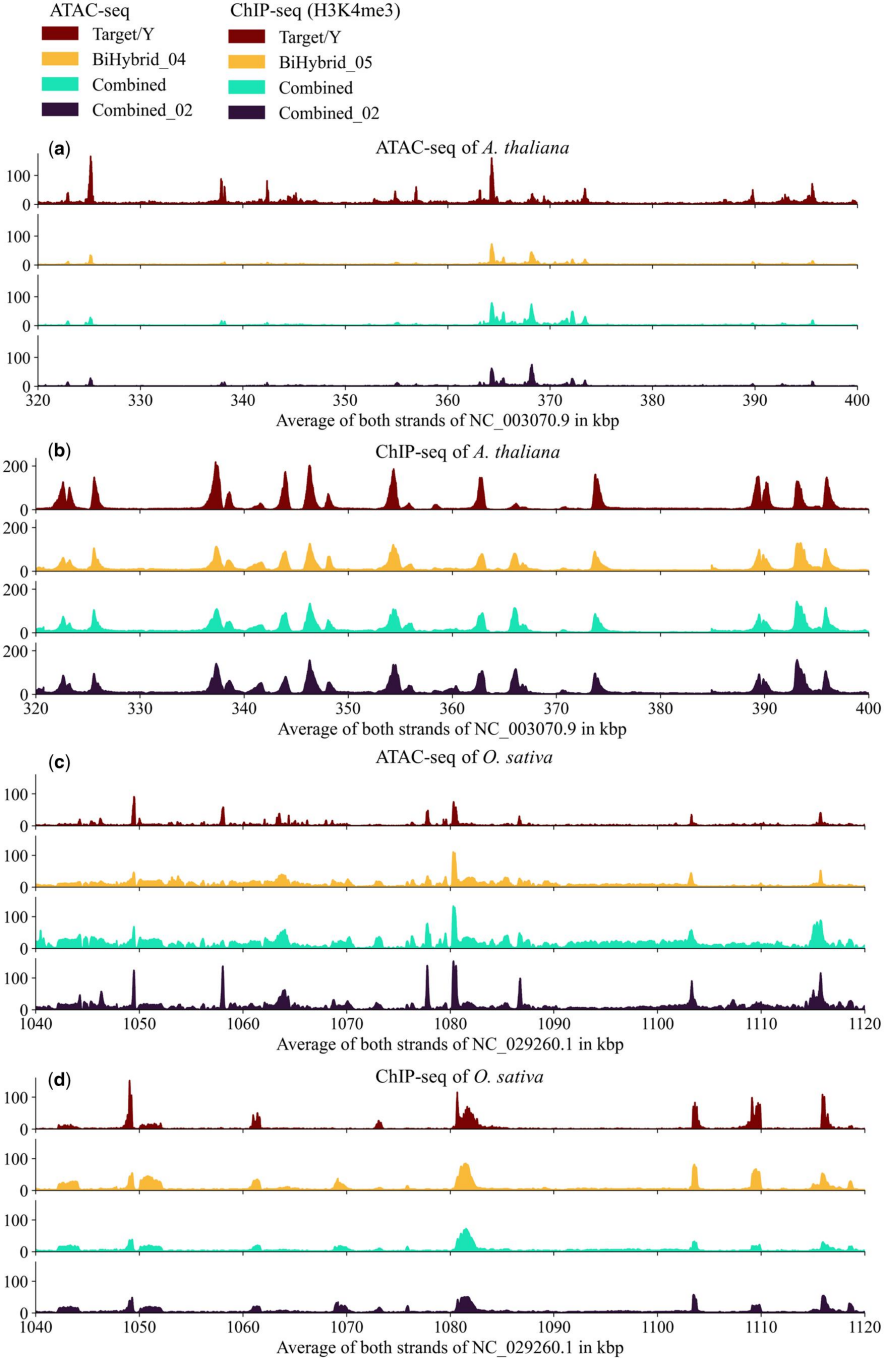
Example predictions of Predmoter. Example regions comparing Predmoter’s unsmoothed predictions to experimental data (target/Y) for the test species (a, b) *A. thaliana* and (c, d) *O. sativa* in 5’ to 3’ direction are depicted. The plots (a) and (c) show the ATAC-seq read coverage per bp, the plots (b) and (d) show ChIP-seq (H3K4me3) read coverage per bp.

To get a more detailed insight into the models’ predictions, zoomed-in example predictions of the BiHybrid_04, BiHybrid_05, Combined and Combined_02 were examined (Fig. 7). The regions were manually selected to present examples for regions with varying levels of prediction quality. By this, we aimed at gaining a deeper understanding of the predictions beyond the quality control using global statistical metrics.

The experimental and predicted peaks showed a common pattern of the ATAC-seq peaks around the TSS overlapping the 5’ UTR. They were usually flanked by a H3K4me3 peak downstream of the TSS. Occasionally the ATAC-seq peak was observed between two histone ChIP-seq peaks, one downstream of the TSS and one upstream of the ATAC-seq peak (Fig. 7). The ATAC-seq predictions for *A.thaliana* showed missing peaks in a few regions compared to the experimental data and in general a lower read coverage (Fig. 7a). The ChIP-seq predictions showed no visible outliers compared to the experimental data (Fig. 7b). All ATAC-seq models examined predicted a higher background noise for *O.sativa* than is present in the experimental data (Fig. 7c). However, the BiHybrid_04 and the Combined model predicted most distinct ATAC-seq peaks in the depicted region, the Combined_02 model predicted all. All models predicted two H3K4me3 ChIP-seq peaks and one ATAC-seq peak for *O.sativa* around 1070 and 1075 kbp that weren’t supported by the experimental data (Fig. 7c and d). The other predicted peaks in the region were at the same position as the experimental peaks.

Predmoter showed a positive linear correlation between inference times and genome length (Supplementary Subsection S1.4, available at *Bioinformatics Advances* online). Inference took longer the more NGS datasets were predicted simultaneously. Predmoter took 2.84 minutes to predict ATAC- and ChIP-seq data together for *A. thaliana*. For *O. sativa* inference took 11.21 minutes.

## 4 Discussion

The identification of CREs is crucial in any attempts to reconstruct gene regulatory networks. In complex genomes, knowledge is mostly concentrated on coding sequences. Studies focusing on the complex genetic mechanisms behind gene regulation fall behind. The high costs and time investments needed to create ATAC- or ChIP-seq libraries are barriers in the way to unravel the natural variation of gene regulation, especially in non-model plants. We developed Predmoter, a low-threshold, fast and precise DNN that uses the target DNA sequence as input and outputs predicted ATAC-seq and ChIP-seq coverage in human-readable format.

Predmoter used both the positive and negative strand as the model’s input. The ATAC- and ChIP-seq read coverage information was also added to both strands (see Section 2.2). The advantages were that open chromatin and closed chromatin regions always apply to both strands, so the addition to both strands allowed for built-in data augmentation. The model benefited from the BiLSTM layers extra information (Fig. 4), as they allowed the network to anticipate a gene region when predicting a promoter (Schuster and Paliwal 1997). Also, the bidirectional interpretation of the data was an appropriate inductive bias, given that Predmoter used unstranded data. Even though batch normalization eliminates the need for dropout layers in some cases (Ioffe and Szegedy 2015), adding one dropout layer with a dropout probability of 30% to Predmoter boosted the predictions (Fig. 4). The predictions were improved for the ChIP-seq data when predicting both datasets together (Fig. 4). The subsequent slight drop-off in the Combined model’s ATAC-seq predictions could be a result of the network having around 20% more ChIP-seq data than ATAC-seq data available to training on (Fig. 3a). The network was skewing just lightly to the larger dataset, at least when looking solely at the Pearson correlation coefficients (Fig. 4). This difference was lessened by adding more data to the training set. The Combined_02 model’s predictions were the closest to the target data for both NGS datasets (Fig. 4). Its training set only contained around 9% more ChIP-seq than ATAC-seq data (Fig. 3a), as well as 5.28% percent more ChIP-seq peaks than ATAC-seq peaks instead of 5.98% (see Supplementary Table S8). These results suggest that Predmoter’s multi label predictions improve by lowering the difference in abundance between the labels/target data, especially between the positive data, i.e. the peaks.

During leave-one-out cross validation, the two models using the alga species *B.natans* and *C.reinhardtii* as validation set stood out for reaching the lowest Pearson’s *r* values (Fig. 4). When inspecting the average read coverage around the TSS for these two species (Fig. 1), especially *C.reinhardtii’*s amplitude position and shape didn’t quite match the data from other species. It might be beneficial to exclude alga species in the future until enough data becomes publicly available to train a dedicated alga model. Both intra-species prediction models achieved higher Pearson’s *r* values than the best cross-species prediction model, the Combined_02 model (Fig. 4). However, the cross-species prediction validation and test metrics show what predictive quality one can expect when predicting on a species not included in the training set. In comparison, intra-species prediction models don’t have to generalize to the same degree, complicating inferring and possibly lowering the predictive quality for a new species.

When using the average coverage of predictions around the TSS region (Fig. 5b) to infer the cutoffs for peak calling, the resulting *F*_1_ scores were high for the ChIP-seq predictions of both test species; the best scores were 0.7388 and 0.7358 for *A.thaliana* and *O.sativa*, respectively (Fig. 6a). The best *F*_1_ scores for ATAC-seq were lower with 0.2162 and 0.5152. The lower *F*_1_ score for the *A.thaliana* ATAC-seq predictions was found to be a matter of thresholding; the precision-recall curve resulted in a AUPRC of 0.512 (Fig. 6b). All AUPRC values exceeded their baseline. The baseline AUPRC is defined as the fraction of positives in the training set (Saito and Rehmsmeier 2015), here the peaks. The best model, the Combined_02 model, exceeded it’s the baseline ATAC-seq AUPRC of 0.0699 by around 0.44 for both test species and the baseline ChIP-seq AUPRC of 0.1227 by 0.7 for *A.thaliana* and 0.6633 for *O.sativa* (Fig. 6b). These results highlight Predmoter’s predictive strength, which can be even further improved by smoothing the original predictions (Fig. 6c).

Nevertheless, Predmoter performs best on ChIP-seq data. The Pearson’s *r* and AUPRC values for the ChIP-seq predictions of all models were higher than the ones for the ATAC-seq predictions (Figs 4–6). H3K4me3 peaks mostly appear in 1000 to 2000 kbp around the TSS including highly conserved gene regions (Santos-Rosa *et al.* 2002, Benayoun *et al.* 2014). Even though CREs were shown to be highly conserved within and among plant species, also between monocots and dicots (Yamamoto *et al.* 2007, Lu *et al.* 2019), they exhibit heterogeneity. The TATA-box, for example, a core promoter element characterized by repeating T and A base pairs (Lifton *et al.* 1978), was found to be present in 16%–22% of core promoters in eight plant species, in 18% of the *A.thaliana* and *O.sativa* core promoters (Kumari and Ware 2013). Therefore, the H3K4me3 peaks were probably easier to learn for the network. This could be supported by the models training only on ChIP-seq data or both datasets also reached their highest validation Pearson correlation coefficient faster (see Supplementary Table S5). The percentage of ChIP-seq peaks in the training set, around 12%, was also higher than the ATAC-seq peak percentage of around 6%–7% (see Supplementary Table S8). This imbalance probably also contributed to the ChIP-seq predictions being closer to the target data than the ATAC-seq predictions.

Looking into the predictions up close allowed us to inspect why some peaks might not get predicted (Fig. 7). The ATAC-seq predictions for *A.thaliana* showed lower read coverage (Figs 5b and 7). The cause might be the in general lower read coverage of the dicot training data in comparison to the monocots (Fig. 1b) or rather the 4% less peaks in the dicot training data in early development stages, in later development stages 2.4% less (see Supplementary Table S8). This might cause higher predicted read coverage for monocots and therefore easier to distinguish peaks. Moreover, predicted peaks in regions missing experimentally verified peaks might appear, because the observed most common pattern was an ATAC-seq peak upstream of a H3K4me3 peak (Fig. 7). The network was likely trying to adhere to that pattern even if the target data did not support it. Another reason for not predicting experimentally verified peaks or predicting peaks in regions where there are none in the experimental data could be the incompleteness of the experimental data. The experimental data originated from different tissues and was treated differently as well (see Supplementary Table S3). The *A. thaliana* ATAC-seq data for example used DNA extracted from leaves (Lu *et al.* 2019) and roots (Maher *et al.* 2018), while the ChIP-seq data used DNA from whole seedlings under cold treatment and after recovery (Xi *et al.* 2020) and an unknown tissue/treatment. Not all genes are always active in every tissue. The choice of tissues and environmental influences can influence the chromatin makeup of the plants’ DNA. Hence, the experimental data shown was not the ground truth. With the currently publicly available, high-quality data for ATAC-seq and H3K4me3 ChIP-seq, the possibility of using as many tissues or treatments as possible to train on or even create dedicated models to specific plant tissues like roots is not yet feasible.

Since ATAC-seq and H3K4me3 ChIP-seq peaks were seen in this study to be close to each other but only partially overlap, other NGS data showing a more similar pattern to ATAC-seq data could improve the predictions for the ACRs. The nearest options would be DNase-seq (Crawford *et al.* 2006) or FAIRE-seq (Giresi *et al.* 2007). Both are less sensitive than ATAC-seq. Another option could be MNase-defined cistrome-Occupancy Analysis (MOA-seq), a high-resolution, high-throughput, and genome-wide strategy to globally identify putative TF-binding sites within ACRs (Savadel *et al.* 2021). The only hindrance would again be the publicly available high-quality data. For example, MOA-seq is too recent to have large amounts of existing published data. Additional ChIP-seq data, like H3K4me1, H3K27ac or H3K27me3, marking enhancers (Heintzman *et al.* 2009, Creyghton *et al.* 2010, Rada-Iglesias *et al.* 2010) or H3K4me2 marking inactive genes (Santos-Rosa *et al.* 2002) could be utilized as well. Weighting the monocot and dicot data as well as the ATAC- and ChIP-seq data to combat overfitting towards a domain or NGS dataset could improve the predictions. Also, a method of normalizing the NGS read coverage without relying on experimental input data could help the network to focus more on peak positions instead of peak amplitudes. Finally, by incorporating peak caller results like from MACS (Zhang *et al.* 2008) into the predictive process of Predmoter, the option of a binary classification model could be added. DL was already used with ATAC-seq data and MACS2 to predict regulatory factor binding activity (Hiranuma *et al.* 2017), to predict enhancers (Thibodeau *et al.* 2018), to optimize ATAC-seq peak calling (Hentges *et al.* 2022) or to predict transcription-factor binding on a genomic scale (Cazares *et al.* 2023). A drawback to using a peak caller would be the introduction of another abstraction level by using the output of another tool/algorithm. In general, more ATAC-seq data from a wider variety of species and tissues would likely improve Predmoter’s predictions more than additional NGS data, since the additional ATAC-seq data used to train the Combined_02 model likely caused it to outperform the Combined model (Fig. 4).

We are aware that Predmoter is strongly limited by the quality and abundance of ATAC- and ChIP-seq data. However, our framework allows for easy retraining with additional high-quality NGS data. This also includes re-training with selected datasets for tissue- or condition specific treatments. In conclusion, Predmoter will help identifying CREs and so gaining further insight into gene regulatory networks in plants.

## Supplementary Material

vbae074_Supplementary_Data

## Data Availability

The datasets in this article were derived from sources in the public domain as listed in section S1.1, Tables S2 and S3.

## References

[vbae074-B1] Andersson R , Gebhard C, Miguel-Escalada I et al An atlas of active enhancers across human cell types and tissues. Nature 2014;507:455–61. 24670763 10.1038/nature12787 PMC5215096

[vbae074-B2] Andrews S. *FastQC A Quality Control tool for High Throughput Sequence Data*, 2010. http://www.bioinformatics.babraham.ac.uk/projects/fastqc.

[vbae074-B3] Avsec Ž , Agarwal V, Visentin D et al Effective gene expression prediction from sequence by integrating long-range interactions. Nat Methods 2021;18:1196–203. 34608324 10.1038/s41592-021-01252-x PMC8490152

[vbae074-B4] Banerji J , Rusconi S, Schaffner W. Expression of a β-Globin gene is enhanced by remote SV40 DNA sequences. Cell 1981;27:299–308. 6277502 10.1016/0092-8674(81)90413-x

[vbae074-B5] Benayoun BA , Pollina EA, Ucar D et al H3K4me3 breadth is linked to cell identity and transcriptional consistency. Cell 2014;158:673–88. 25083876 10.1016/j.cell.2014.06.027 PMC4137894

[vbae074-B6] Bolger AM , Lohse M, Usadel B. Trimmomatic: a flexible trimmer for illumina sequence data. Bioinformatics 2014;30:2114–20. 24695404 10.1093/bioinformatics/btu170 PMC4103590

[vbae074-B7] Broad Institute ed. Picard Toolkit. *Broad Institute*, 2019. https://github.com/broadinstitute/picard.

[vbae074-B8] Buenrostro JD , Giresi PG, Zaba LC et al Transposition of native chromatin for fast and sensitive epigenomic profiling of open chromatin, DNA-binding proteins and nucleosome position. Nat Methods 2013;10:1213–8. 24097267 10.1038/nmeth.2688 PMC3959825

[vbae074-B9] Cazares TA , Rizvi FW, Iyer B et al maxATAC: genome-scale transcription-factor binding prediction from ATAC-seq with deep neural networks. PLoS Comput Biol 2023;19:e1010863. 36719906 10.1371/journal.pcbi.1010863 PMC9917285

[vbae074-B10] Chen Y , Gao Y, Zhou H et al AthEDL: identifying enhancers in Arabidopsis thaliana using an attention-based deep learning method. Cbio 2022;17:531–40.

[vbae074-B11] Cockerill PN. Structure and function of active chromatin and DNase I hypersensitive sites. FEBS J 2011;278:2182–210.21501387

[vbae074-B12] Crawford GE , Holt IE, Whittle J et al Genome-wide mapping of DNase hypersensitive sites using massively parallel signature sequencing (MPSS). Genome Res 2006;16:123–31. 16344561 10.1101/gr.4074106 PMC1356136

[vbae074-B13] Creyghton MP , Cheng AW, Welstead GG et al Histone H3K27ac separates active from poised enhancers and predicts developmental state. Proc Natl Acad Sci USA 2010;107:21931–6. 21106759 10.1073/pnas.1016071107 PMC3003124

[vbae074-B14] Danecek P , Bonfield JK, Liddle J et al Twelve years of SAMtools and BCFtools. Gigascience 2021;10:giab008. 10.1093/GIGASCIENCE/GIAB008 PMC7931819 33590861

[vbae074-B15] Dao LTM , Galindo-Albarrán AO, Castro-Mondragon JA et al Genome-wide characterization of mammalian promoters with distal enhancer functions. Nat Genet 2017;49:1073–81. 28581502 10.1038/ng.3884

[vbae074-B16] Diao Y , Fang R, Li B et al A tiling-deletion-based genetic screen for cis-regulatory element identification in mammalian cells. Nat Methods 2017;14:629–35. 28417999 10.1038/nmeth.4264 PMC5490986

[vbae074-B17] Dynan WS , Tjian R. Control of eukaryotic messenger RNA synthesis by sequence-specific DNA-binding proteins. Nature 1985;316:774–8. 4041012 10.1038/316774a0

[vbae074-B18] Engreitz JM , Haines JE, Perez EM et al Local regulation of gene expression by lncRNA promoters, transcription and splicing. Nature 2016;539:452–5. 27783602 10.1038/nature20149 PMC6853796

[vbae074-B19] Epstein W , Beckwith JR. Regulation of gene expression. Annu Rev Biochem 1968;37:411–36.

[vbae074-B20] Ewels P , Magnusson M, Lundin S et al MultiQC: summarize analysis results for multiple tools and samples in a single report. Bioinformatics 2016;32:3047–8. 27312411 10.1093/bioinformatics/btw354 PMC5039924

[vbae074-B21] Falcon W. Pytorch Lightning, 2019. https://github.com/Lightning-AI/pytorch-lightning.

[vbae074-B22] Gao Y , Chen Y, Feng H et al RicENN: prediction of rice enhancers with neural network based on DNA sequences. Interdiscip Sci 2022;14:555–65. 35190950 10.1007/s12539-022-00503-5

[vbae074-B23] Giresi PG , Kim J, McDaniell RM et al FAIRE (formaldehyde-assisted isolation of regulatory elements) isolates active regulatory elements from human chromatin. Genome Res 2007;17:877–85. 17179217 10.1101/gr.5533506 PMC1891346

[vbae074-B24] Glorot X , Bordes A, Bengio Y. Deep sparse rectifier neural networks. In: *Proceedings of the Fourteenth International Conference on Artificial Intelligence and Statistics*. PMLR. JMLR Workshop and Conference Proceedings, Fort Lauderdale, FL, USA, 2011, 315–23.

[vbae074-B25] Gross DS , Garrard WT. Nuclease hypersensitive sites in chromatin. Annu Rev Biochem 1988;57:159–97. 3052270 10.1146/annurev.bi.57.070188.001111

[vbae074-B26] Heintzman ND , Hon GC, Hawkins RD et al Histone modifications at human enhancers reflect global cell-type-specific gene expression. Nature 2009;459:108–12. 19295514 10.1038/nature07829 PMC2910248

[vbae074-B27] Hentges LD , Sergeant MJ, Cole CB et al LanceOtron: a deep learning peak caller for genome sequencing experiments. Bioinformatics 2022;38:4255–63. 35866989 10.1093/bioinformatics/btac525 PMC9477537

[vbae074-B28] Hiranuma N , Lundberg S, Lee S-I. DeepATAC: a deep-learning method to predict regulatory factor binding activity from ATAC-seq signals. *bioRxiv*, 10.1101/172767, 2017, preprint: not peer reviewed.

[vbae074-B29] Hochreiter S , Schmidhuber J. Long Short-Term memory. Neural Comput 1997;9:1735–80. 9377276 10.1162/neco.1997.9.8.1735

[vbae074-B30] Holst F , Bolger A, Günther C et al Helixer–de novo Prediction of Primary Eukaryotic Gene Models Combining Deep Learning and a Hidden Markov Model. *bioRxiv*, 10.1101/2023.02.06.527280, 2023, preprint: not peer reviewed. PMC13076211 41286201

[vbae074-B31] Hong J , Gao R, Yang Y. CrepHAN: cross-species prediction of enhancers by using hierarchical attention networks. Bioinformatics 2021;37:3436–43. 33978703 10.1093/bioinformatics/btab349

[vbae074-B32] Ioffe S , Szegedy C. Batch normalization: accelerating deep network training by reducing internal covariate shift. In: *Proceedings of the 32nd International Conference on Machine Learning*. Lille, France: PMLR, 2015, 448–56.

[vbae074-B33] Ippen K , Miller JH, Scaife J et al New controlling element in the Lac operon of *E. coli*. Nature 1968;217:825–7. 4867974 10.1038/217825a0

[vbae074-B34] Jacob F , Ullman A, Monod J. Le promoteur, élément génétique nécessaire à l’expression d’un opéron. CR Acad Sci(Paris) 1964;258:3125–8. 14143651

[vbae074-B35] Johnson DS , Mortazavi A, Myers RM et al Genome-wide mapping of in vivo protein-DNA interactions. Science 2007;316:1497–502. 17540862 10.1126/science.1141319

[vbae074-B36] Kim J , Shujaat M, Tayara H. iProm-Zea: a two-layer model to identify plant promoters and their types using convolutional neural network. Genomics 2022;114:110384. 35533969 10.1016/j.ygeno.2022.110384

[vbae074-B37] Kim J , Zeller KI, Wang Y et al Evaluation of myc E-Box phylogenetic footprints in glycolytic genes by chromatin immunoprecipitation assays. Mol Cell Biol 2004;24:5923–36. 15199147 10.1128/MCB.24.13.5923-5936.2004 PMC480875

[vbae074-B38] Kim TK , Hemberg M, Gray JM et al Widespread transcription at neuronal activity-regulated enhancers. Nature 2010;465:182–7. 20393465 10.1038/nature09033 PMC3020079

[vbae074-B39] Kingma DP , Ba JL. Adam: a method for stochastic optimization. In: *3rd International Conference on Learning Representations, ICLR 2015 - Conference Track Proceedings*, ArXiv, San Diego, CA, USA, 2014. 10.48550/arxiv.1412.6980

[vbae074-B40] Kumari S , Ware D. Genome-Wide computational prediction and analysis of core promoter elements across plant monocots and dicots. PLoS One 2013;8:e79011. 24205361 10.1371/journal.pone.0079011 PMC3812177

[vbae074-B41] LeCun Y , Bengio Y. Convolutional networks for images, speech, and time-series. In: Arbib MA (ed.), *The Handbook of Brain Theory and Neural Networks*. Cambridge, MA, USA: MIT Press, 1995, 255–8.

[vbae074-B42] LeCun Y , Boser B, Denker J et al Handwritten digit recognition with a Back-Propagation network. Adv Neural Inf Process Syst 1989;2:396–404.

[vbae074-B43] Li J , Wu Z, Lin W et al iEnhancer-ELM: improve enhancer identification by extracting position-related multiscale contextual information based on enhancer language models. Bioinform Adv 2023;3:vbad043. 37113248 10.1093/bioadv/vbad043 PMC10125906

[vbae074-B44] Lifton RP , Goldberg ML, Karp RW et al The organization of the histone genes in Drosophila melanogaster: functional and evolutionary implications. Cold Spring Harb Symp Quant Biol 1978;42(Pt 2):1047–51. 98262 10.1101/sqb.1978.042.01.105

[vbae074-B45] Lu Z , Marand AP, Ricci WA et al The prevalence, evolution and chromatin signatures of plant regulatory elements. Nat Plants 2019;5:1250–9. 31740772 10.1038/s41477-019-0548-z

[vbae074-B46] Maher KA , Bajic M, Kajala K et al Profiling of accessible chromatin regions across multiple plant species and cell types reveals common gene regulatory principles and new control modules. Plant Cell 2018;30:15–36. 29229750 10.1105/tpc.17.00581 PMC5810565

[vbae074-B47] McInnes L , Healy J, Melville J. UMAP: uniform manifold approximation and projection for dimension reduction. *J Open Source Softw* 2018;3:861. 10.21105/joss.00861.

[vbae074-B48] Md V , Misra S, Li H et al Efficient architecture-aware acceleration of BWA-MEM for multicore systems.In: *Proceedings - 2019 IEEE 33rd International Parallel and Distributed Processing Symposium, IPDPS 2019,* Rio de Janeiro, Brazil, 2019, 314–24.

[vbae074-B49] Osmala M , Lähdesmäki H. Enhancer prediction in the human genome by probabilistic modelling of the chromatin feature patterns. BMC Bioinformatics 2020;21:317. 32689977 10.1186/s12859-020-03621-3 PMC7370432

[vbae074-B50] Oubounyt M , Louadi Z, Tayara H et al Deepromoter: robust promoter predictor using deep learning. Front Genet 2019;10:286. 31024615 10.3389/fgene.2019.00286 PMC6460014

[vbae074-B51] Paszke A , Gross S, Massa F et al PyTorch: an imperative style, high-performance deep learning library. Adv Neural Inf Process Syst 2019;32:1–12. 10.48550/arxiv.1912.01703

[vbae074-B52] Pedregosa F , Varoquaux G, Gramfort A et al Scikit-learn: machine learning in python. J Mach Learn Res 2011;12:2825–30.

[vbae074-B53] Rada-Iglesias A , Bajpai R, Swigut T et al A unique chromatin signature uncovers early developmental enhancers in humans. Nature 2010;470:279–83. 21160473 10.1038/nature09692 PMC4445674

[vbae074-B54] Ramírez F , Ryan DP, Grüning B et al deepTools2: a next generation web server for deep-sequencing data analysis. Nucleic Acids Res 2016;44:W160–5. 27079975 10.1093/nar/gkw257 PMC4987876

[vbae074-B55] Robertson G , Hirst M, Bainbridge M et al Genome-wide profiles of STAT1 DNA association using chromatin immunoprecipitation and massively parallel sequencing. Nat Methods 2007;4:651–7. 17558387 10.1038/nmeth1068

[vbae074-B56] Russell SJ , Norvig P. Artificial Intelligence: A Modern Approach. Global Edition. Upper Saddle River, New Jersey: PEV, Pearson Education, Inc., 2016.

[vbae074-B57] Saito T , Rehmsmeier M. The Precision-Recall plot is more informative than the ROC plot when evaluating binary classifiers on imbalanced datasets. PLoS One 2015;10:e0118432. 25738806 10.1371/journal.pone.0118432 PMC4349800

[vbae074-B58] Santa Fd , Barozzi I, Mietton F et al A large fraction of extragenic RNA pol II transcription sites overlap enhancers. PLoS Biol 2010;8:e1000384. 20485488 10.1371/journal.pbio.1000384 PMC2867938

[vbae074-B59] Santos-Rosa H , Schneider R, Bannister AJ et al Active genes are tri-methylated at K4 of histone H3. Nature 2002;419:407–11. 12353038 10.1038/nature01080

[vbae074-B60] Savadel SD , Hartwig T, Turpin ZM et al The native cistrome and sequence motif families of the maize ear. PLoS Genet 2021;17:e1009689. 34383745 10.1371/journal.pgen.1009689 PMC8360572

[vbae074-B61] Schulz H , Behnke S. Deep learning: layer-wise learning of feature hierarchies. KI—Kunstliche Intelligenz 2012;26:357–63.

[vbae074-B62] Schuster M , Paliwal KK. Bidirectional recurrent neural networks. IEEE Trans Signal Process 1997;45:2673–81.

[vbae074-B63] Shujaat M , Lee SB, Tayara H et al Cr-Prom: a convolutional neural Network-Based model for the prediction of rice promoters. IEEE Access 2021;9:81485–91.

[vbae074-B64] Song L , Zhang Z, Grasfeder LL et al Open chromatin defined by DNaseI and FAIRE identifies regulatory elements that shape cell-type identity. Genome Res 2011;21:1757–67. 21750106 10.1101/gr.121541.111 PMC3202292

[vbae074-B65] Stiehler F , Steinborn M, Scholz S et al Helixer: cross-species gene annotation of large eukaryotic genomes using deep learning. Bioinformatics 2021;36:5291–8. 33325516 10.1093/bioinformatics/btaa1044 PMC8016489

[vbae074-B66] Struhl K. Yeast transcriptional regulatory mechanisms. Annu Rev Genet 1995;29:651–74. 8825489 10.1146/annurev.ge.29.120195.003251

[vbae074-B67] Thibodeau A , Uyar A, Khetan S et al A neural network based model effectively predicts enhancers from clinical ATAC-seq samples. Sci Rep 2018;8:16048–15. 30375457 10.1038/s41598-018-34420-9 PMC6207744

[vbae074-B68] Wang Y , Peng Q, Mou X et al A successful hybrid deep learning model aiming at promoter identification. BMC Bioinformatics 2022;23:206–20. 35641900 10.1186/s12859-022-04735-6 PMC9158169

[vbae074-B69] Xi Y , Park SR, Kim DH et al Transcriptome and epigenome analyses of vernalization in Arabidopsis thaliana. Plant J 2020;103:1490–502. 32412129 10.1111/tpj.14817 PMC7434698

[vbae074-B70] Yamamoto YY , Ichida H, Matsui M et al Identification of plant promoter constituents by analysis of local distribution of short sequences. BMC Genomics 2007;8:67–23. 17346352 10.1186/1471-2164-8-67 PMC1832190

[vbae074-B71] Yuan H , Kelley DR. scBasset: sequence-based modeling of single-cell ATAC-seq using convolutional neural networks. Nat Methods 2022;19:1088–96. 35941239 10.1038/s41592-022-01562-8

[vbae074-B72] Zhang Y , Liu T, Meyer CA et al Model-based analysis of ChIP-Seq (MACS). Genome Biol 2008;9:R137–9. 18798982 10.1186/gb-2008-9-9-r137 PMC2592715

[vbae074-B73] Zheng L , McMullen MD, Bauer E et al Prolonged expression of the BX1 signature enzyme is associated with a recombination hotspot in the benzoxazinoid gene cluster in Zea mays. J Exp Bot 2015;66:3917–30. 25969552 10.1093/jxb/erv192 PMC4473990

[vbae074-B74] Zhu Y , Li F, Xiang D et al Computational identification of eukaryotic promoters based on cascaded deep capsule neural networks. Brief Bioinform 2021;22:bbaa299. 33227813 10.1093/bib/bbaa299 PMC8522485

