# Supplementary material for: Predmoter—cross-species prediction of plant promoter and enhancer regions
Source: Bioinform Adv. 2024 May 24;4(1):vbae074. doi: 10.1093/bioadv/vbae074 (PMC11150885; doi:10.1093/bioadv/vbae074)
Supplement: vbae074_Supplementary_Data [file vbae074_supplementary_data.pdf]

Supplemental material for

Predmoter - Cross-species prediction of plant promoter and enhancer associated NGS data

---

Table of Contents

S1 Supplemental Methods **2**

S1.1 Data 2

S1.2 Architecture 2

S1.3 Training details 3

S1.4 Benchmarking 3

S1.5 Figure creation 3

S2 Supplemental Figures **4**

S2.1 Alternative Figures 4

S2.2 Benchmarking 5

S3 Supplemental Tables **6**

S3.1 Data 6

S3.2 Training parameters 10

S3.3 Tabular results 10

S3.4 Peak statistics 12

References **13**

S1 Supplemental Methods

S1.1 Data

The plant genomes were acquired from NCBI's RefSeq or GenBank. The ATAC- and ChIP-seq data is publicly available data acquired from the NCBI's SRA (Tab. S2). The different plant tissues and treatments used in the ATAC- and ChIP-seq experiments are listed if available (Tab. S3). The datasets of the three species *Actinidia chinensis*, *Panicum miliaceum*, and *Sorghum bicolor*, as well as the ChIP-seq dataset of *Marchantia polymorpha* were only used in later development stages.

S1.2 Architecture

Predmoter uses custom padding formulas to ensure sequence length divisibility and tensor shape consistency. The padding formulas are adapted from the PyTorch documentation. First, the formula to calculate the output sequence length (L_{out}) of a one-dimensional convolution, by using the input variables: padding, dilation, kernel size, stride, and the input sequence length (L_{in}), was rearranged (Equation S1). The outer brackets indicate to round the result down (https://pytorch.org/docs/stable/generated/torch.nn.Conv1d.html).

$$L_{out} = \left\lfloor \frac{L_{in} + 2*padding - dilation*(kernelsize - 1) - 1}{stride} + 1 \right\rfloor \quad \text{(S1)}$$

The initial input sequence length, default is 21384 bp, needs to be divisible by the chosen stride (referred to as step in Predmoter). For multiple convolutional layers the sequence length needs to be divisible by the chosen stride to the power of the chosen number of convolutional layers. Since the convolutional layers don't evenly divide the given sequence length, i.e., a sequence length of 21384 and a stride of 2 should result in an output sequence length of 10692, a padding formula was calculated to ensure even division. The result of this formula is the number of zeros added to both sides of the input. For example, padding of 3 would result in 3 zeros getting added to the one-dimensional input tensor on both sides. Padding is calculated using the variables: output sequence length (L_{out}), dilation, kernel size, stride, and the input sequence length (L_{in}) of the one-dimensional convolutional layer, where the desired L_{out} is: $L_{out} = \frac{L_{in}}{stride}$. The outer brackets indicate to round the result up.

$$padding = \left\lceil \frac{(L_{out} - 1)*stride - L_{in} + dilation*(kernelsize - 1) + 1}{2} \right\rceil \quad \text{(S2)}$$

Second, the formula to calculate the output sequence length (L_{out}) of a one-dimensional transposed convolution, by using the input variables padding, output padding, dilation, kernel size, stride, and the input sequence length (L_{in}) (https://pytorch.org/docs/stable/generated/torch.nn.ConvTranspose1d.html) was rearranged (Equation S3).

$$L_{out} = (L_{in} - 1) * stride - 2 * padding + dilation * (kernelsize - 1) + output\_padding + 1 \quad \text{(S3)}$$

Padding is calculated using the variables: output sequence length (L_{out}), output padding, dilation, kernel size, stride, and the input sequence length (L_{in}), where the desired L_{out} is: $L_{out} = L_{in} * \mathrm{stride}$ (Equation S4). The outer brackets indicate to round the result down. In contrast to the one-dimensional convolution, the amount of zero-padding applied to both sides of the input tensor is calculated via this formula: $dilation * (kernelsize - 1) - padding$. For example, if the result of the custom padding formula is 6, the kernel size is 18 and the dilation is 1, then 11 zeros will be added to both sides of the input tensor. The additional variable output padding is only used to find the correct output shape (Equation S5-S6). It does not add zero-padding to the output tensor. The combination of an even kernel size and a stride of 1 is currently not possible due to a limitation in PyTorch.

$$padding = \left\lfloor \frac{(L_{in} - 1)*stride - L_{out} + dilation*(kernelsize - 1) + output\_padding + 1}{2} \right\rfloor \quad \text{(S4)}$$

where:

$$output\_padding = \begin{cases} 0, & if \quad 2 \mid (kernel\_size + stride) \\ 1, & otherwise \end{cases} \quad \text{(S5)}$$

except $if\ 2 \mid dilation\ and\ 2 \mid kernel\_size$, then:

$$output\_padding = \begin{cases} 1, & if \quad 2 \mid (kernel\_size + stride) \\ 0, & otherwise \end{cases} \quad \text{(S6)}$$

S1.3 Training details

The models were trained and tested on a server with an Intel(R) Xeon(R) CPU E5-2640v4 (Broadwell) @ 2.40 GHz and an Nvidia GeForce GTX 1080 Ti GPU (11 Gb memory). The software versions were CUDA 11.7.1, cuDNN 8.7.0 and Python 3.8.3 (Rossum and Drake 2009). The relevant Python package versions were H5py 3.9.0 (Collette 2013), PyTorch 2.0.1 (Paszke *et al.* 2019), Lightning 2.0.4 (Falcon 2019), Helixer version 0.3.2 (Stiehler *et al.* 2021; Holst *et al.* 2023) and Predmoter version 0.3.2. The exact versions of the other packages used can be found in: https://github.com/weberlab-hhu/Predmoter/blob/main/training_package_versions_freeze.txt. The same server and setup were used for generating the predictions for *Arabidopsis thaliana* and *Oryza sativa*.

Three replicates per model setup were trained utilizing three different seeds: 132709648, 961333724 and 4227086911. The species selection for each model setup is listed in Table 3. The exact training parameters can be found in Table S4. The two inter-species and 25 leave-one-out cross-validation models used the same parameters as the Combined_02 model. All models were trained until convergence, meaning until the listed "stop-quantity" stopped improving for the set number of epochs (patience). The best models, the models with the highest Pearson's r value for the validation set are listed in Table S6. These models were used to compare the target data to the model's predictions for each species from the training, validation, and test set. Testing was performed with four workers/CPUs, one GPU and a batch size of 200. The tabular results are listed in Tables S7 and S8. Predictions on the test set were also generated with four workers/CPUs, one GPU and a batch size of 200. Direct conversion from the h5 output file to a or multiple bigwig files was chosen. The predictions for the test species including the flagged regions are shown in Figure S1.

S1.4 Benchmarking

Benchmarking was performed on a machine with an Intel(R) Xeon(R) CPU W-2125 @ 4.00 GHz and an Nvidia GeForce GTX 1050 Ti GPU (4 Gb memory). The software versions were CUDA 11.5, cuDNN 8.9.5 and Python 3.10.12 (Rossum and Drake 2009). The relevant Python package versions were H5py 3.9.0 (Collette 2013), PyTorch 2.0.1 (Paszke *et al.* 2019), Lightning 2.0.8 (Falcon 2019), Helixer version 0.3.2 (Stiehler *et al.* 2021; Holst *et al.* 2023) and Predmoter version 0.3.2. The exact versions of the other packages used can be found in: https://github.com/weberlab-hhu/Predmoter/blob/main/benchmarking_package_versions_freeze.txt.

Depending on the model used, there is always a slight fluctuation in the prediction and conversion to bigWig or bedGraph files. Two different models BiHybrid_04 and the combined model were used, as predicting two datasets increases the computing time. Three benchmarking figures are shown. The first shows benchmarking Helixer's conversion from fasta to h5 files (Fig. S2). The second shows benchmarking inference and converting these into bigWig and bedGraph files using BiHybrid_04, a model only trained on and able to predict ATAC-seq data (Fig. S3). The final figure shows benchmarking inference and converting these into bigWig and bedGraph files using the combined model trained on and able to predict ATAC- and ChIP-seq data (Fig. S4). Some genome assemblies were highly fragmented, on contig or scaffold level, increasing the number of subsequences. For example, the genome assembly of *Arabidopsis thaliana* wasn't highly fragmented, the genome size being 119.7 Mbp and the number of 21384 bp subsequences of the h5 file created by Helixer was 11202. The genome of *B. natans* was highly fragmented, the genome size being 91.4 Mbp, but the h5 file contained 13390 subsequences. Since inference and conversion to bigWig or bedGraph files is dependent on the amount of data, so the number of subsequences, that was used to quantify the wall time (Fig. S3 & S4).

S1.5 Figure creation

The taxonomy tree in Figure 6 was created with the NCBI's Taxonomy Common Tree application and visualized using iTOL (Letunic and Bork 2021). The heatmaps and coverage plots were created with Matplotlib (Hunter 2007) and Seaborn (Waskom 2021). Jupyter Notebooks (Kluyver *et al.* 2016) detailing the creation of figures and parts of figures respectively can be found at https://github.com/weberlab-hhu/Predmoter/blob/main/visualization.

S2 Supplemental Figures

S2.1 Alternative figures

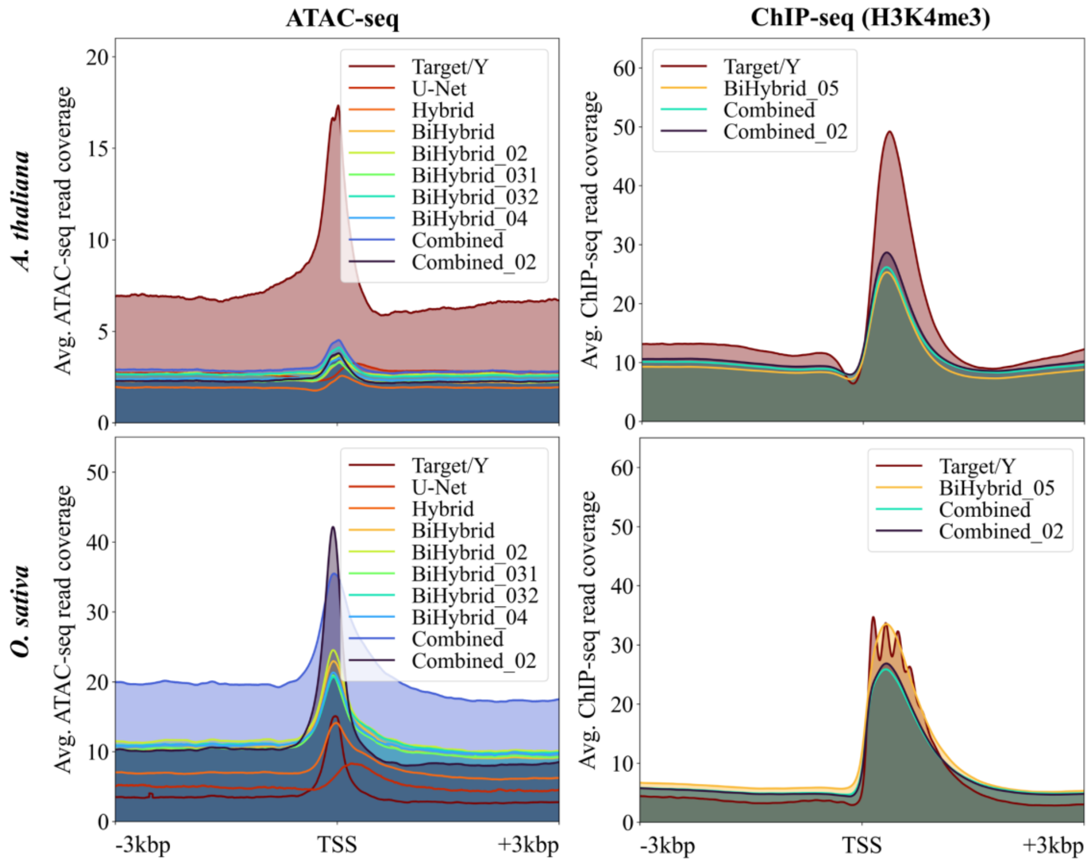

Figure S1: Experimental and predicted ATAC- and ChIP-seq read coverage +/- 3 kbp around the TSS. The average experimental read coverage (target/y) and predicted ATAC- and ChIP-seq read coverage, excluding unplaced scaffolds and non-nuclear sequences, in reads per base pair are shown for *A. thaliana* and *O. sativa*. The predictions of all cross-species models were plotted. Flagged sequences were excluded from the calculations.

S2.2 Benchmarking

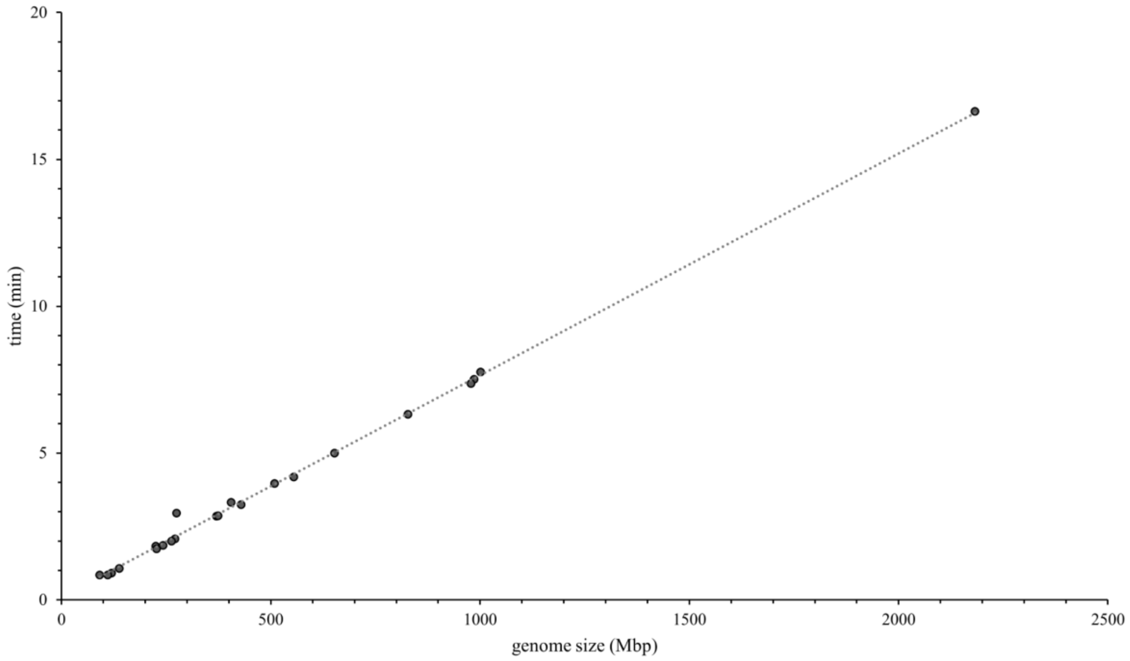

Figure S2: Benchmarking conversion from fasta to h5 file. Helixer's wall time for converting fasta to h5 files in minutes for all the species/genome assemblies used in this study. The gapped genome size including unplaced scaffolds in Mbp is shown on the x-axis. The individual data points and a linear trend line are depicted.

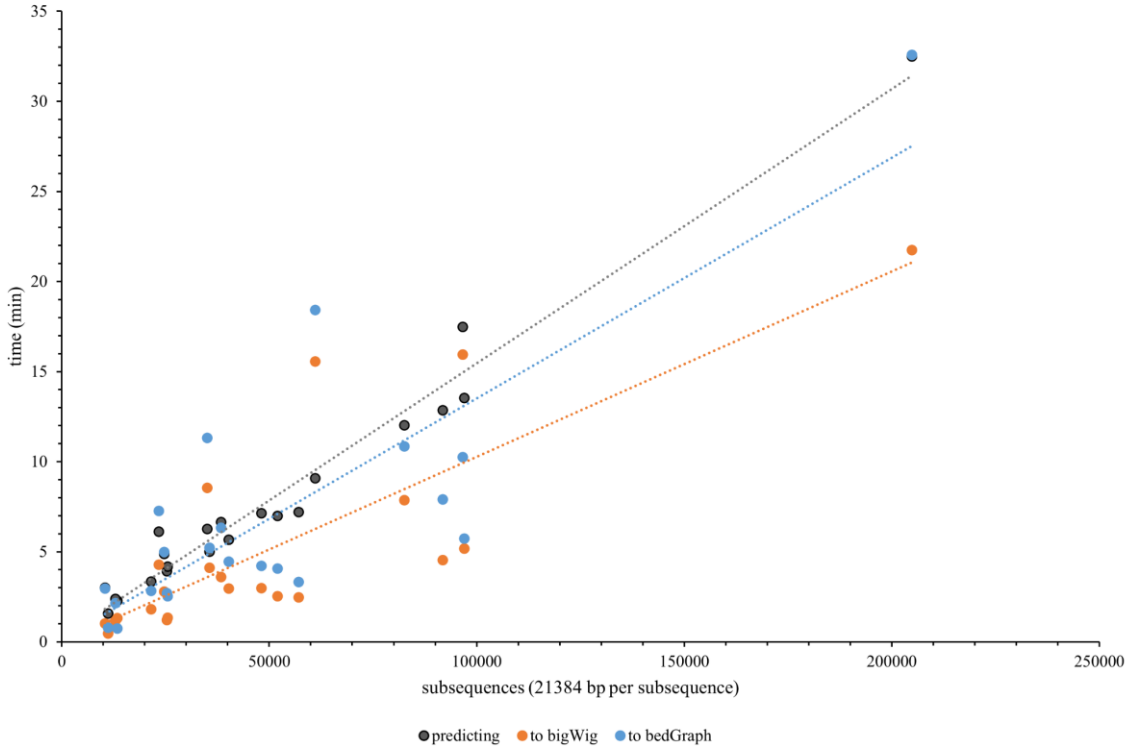

Figure S3: Benchmarking 1. The prediction time (black) and prediction h5 file conversion time to bigWig (orange) and bedGraph (blue) files for ATAC-seq read coverage are depicted. The model used for predicting was the BiHybrid_04 model. The wall time in minutes is shown on the y-axis and the number of subsequences, one subsequence is 21384 bp long, on the x-axis. The individual data points and a linear trend line are displayed.

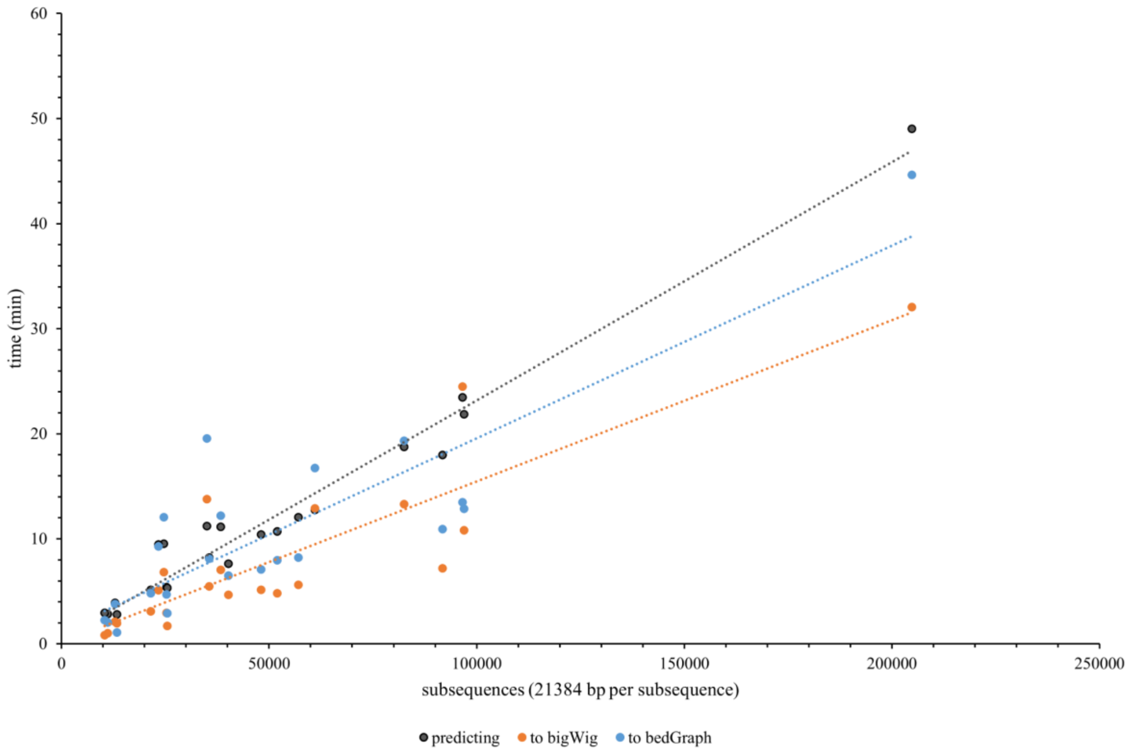

Figure S4: Benchmarking 2. The prediction time (black) and prediction h5 file conversion time to bigWig (orange) and bedGraph (blue) files for ATAC- and ChIP-seq read coverage are depicted. The model used for predicting was the combined model. The wall time in minutes is shown on the y-axis and the number of subsequences, one subsequence is 21384 bp long, on the x-axis. The individual data points and a linear trend line are displayed.

S3 Supplemental Tables

S3.1 Data

Table S1: Nucleotide encoding.

Base	Encoding
C	[1., 0., 0., 0.]
A	[0., 1., 0., 0.]
T	[0., 0., 1., 0.]
G	[0., 0., 0., 1.]
Y	[0.5, 0., 0.5, 0.]
R	[0., 0.5, 0., 0.5]
W	[0., 0.5, 0.5, 0.]
S	[0.5, 0., 0., 0.5]
K	[0., 0., 0.5, 0.5]
M	[0.5, 0.5, 0., 0.]
D	[0., 0.33, 0.33, 0.33]
V	[0.33, 0.33, 0., 0.33]
H	[0.33, 0.33, 0.33, 0.]
B	[0.33, 0., 0.33, 0.33]
N	[0.25, 0.25, 0.25, 0.25]

The bases and possible other notations aside from C, A, T or G and the corresponding one-hot vector encoding used for the input DNA sequence are listed.

Table S2: Data origin.

Species (scientific)	ATAC-seq (SRA and BioProject accessions)	ChIP-seq (SRA and BioProject accessions)	Genome (RefSeq/GenBank accession)	Split

Actinidia chinensis	PRJNA1039995*:<br>SRR26816478<br>SRR26816479	PRJNA1039995*:<br>SRR26816470<br>SRR26816484	GCA_009663005.1	train
Arabidopsis thaliana	PRJNA394532:<br>SRS2357129<br>SRS2357131<br>SRS2357132<br>(Maher *et al.* 2018)<br>PRJNA527732:<br>SRS4500485<br>SRS4500486<br>(Lu *et al.* 2019)	PRJNA408288:<br>SRR6057430<br>SRR6057434<br>PRJNA535479:<br>SRS4672527<br>SRS4672528<br>SRS4672529<br>(Xi *et al.* 2020)	GCF_000001735.4	test
Bigelowiella natans	PRJNA753294:<br>SRS9735275<br>(Marinov *et al.* 2022)		GCA_000320545.1	train
Brachypodium distachyon	PRJNA661629:<br>SRS7327661<br>SRS7327662<br>SRS7327689<br>SRS7327690<br>(An *et al.* 2020)	PRJNA661629:<br>SRS7327667<br>SRS7327668<br>SRS7327669<br>SRS7327670<br>(An *et al.* 2020)	GCF_000005505.3	train
Brassica napus	PRJNA808238:<br>SRS12055968<br>SRS12055970	PRJNA687926:<br>SRS7933167<br>(Li, Li and Wang 2022)	GCF_020379485.1	train
Brassica oleracea		PRJNA687926:<br>SRS7933149<br>(Li, Li and Wang 2022)	GCA_900416815.2	train
Brassica rapa		PRJNA687926:<br>SRS7933147<br>(Li, Li and Wang 2022)	GCA_016163755.1	train
Chlamydomonas reinhardtii		PRJNA681680:<br>SRR13170450	GCF_000002595.2	train
Eragrostis nindensis	PRJNA807505:<br>SRS12036931<br>SRS12036932	PRJNA548367:<br>SRS4948778<br>SRS4948779<br>SRS4948781<br>SRS4948782	GCA_012490785.1	train
Glycine max	PRJNA657378:<br>SRS7209174<br>SRS7209175<br>(Huang *et al.* 2021)	PRJNA753632:<br>SRR15458316<br>SRR15458321<br>(Yung *et al.* 2022)	GCF_000004515.6	train
Malus domestica	PRJNA821644:<br>SRS12449334<br>SRS12449335	PRJNA267727:<br>SRS752518	GCA_916612005.1	train
Marchantia polymorpha	PRJNA597314:<br>SRR10879463<br>SRR10879464	PRJNA1043823*:<br>SRS19609405<br>SRS19609406	GCA_003032435.1	train
Medicago truncatula	PRJNA647765:<br>SRS7054112<br>SRS7054113<br>SRS7054114<br>SRS7054115<br>SRS7054116<br>SRS7054117<br>SRS7054118<br>(Pereira *et al.* 2022)	PRJNA783892:<br>SRS11159582<br>SRS11159583<br>(Jaudal *et al.* 2022)	GCF_003473485.1	val
Oropetium thomaeum	PRJNA807505:<br>SRS12036929<br>SRS12036933<br>SRS12036934<br>SRS12036935		GCA_001182835.1	train
Oryza brachyantha		PRJNA521886:<br>SRS4357813<br>SRS4357820	GCF_000231095.2	train

		SRS4357828		
Oryza sativa	PRJNA751145:<br>SRS9651698<br>SRS9651700<br>SRS9651701<br>SRS9651704<br>SRS9651708	PRJNA386513:<br>SRS2419794<br>SRS2419800<br>SRS2419801	GCF_001433935.1	test
Panicum miliaceum	PRJNA1063172*:<br>SRR27704574<br>SRR27704575		GCA_032594955.1	train
Prunus persica		PRJNA381300:<br>SRS2712226<br>SRS2712231<br>PRJNA589110:<br>SRS5638125<br>SRS5638127<br>SRS5638129	GCF_000346465.2	train
Pyrus x bretschneideri		PRJNA669907:<br>SRS7570511<br>SRS7570512<br>SRS7570517<br>SRS7570518<br>SRS7570519<br>SRS7570520<br>SRS7570521	GCF_019419815.1	train
Sesamum indicum		PRJNA577518:<br>SRS5511999<br>SRS5512000<br>SRS5512001<br>SRS5512002	GCF_000512975.1	train
Setaria italica		PRJNA391551:<br>SRS2307763<br>PRJNA486213:<br>SRS3675865	GCF_000263155.2	train
Solanum lycopersicum	PRJNA850391:<br>SRS13475373<br>(Huang *et al.* 2023)<br>PRJNA937410:<br>SRS16948284<br>SRS16948285<br>SRS16948288	PRJNA624889:<br>SRS6475095<br>SRS6475096<br>SRS6475097	GCF_000188115.5	train
Sorghum bicolor	PRJNA1063172*:<br>SRR27704580<br>SRR27704581		GCF_000003195.3	train
Spirodela polyrhiza	PRJNA527732:<br>SRS4500499<br>SRS4500501<br>(Lu *et al.* 2019)	PRJNA527732:<br>SRS4500430<br>(Lu *et al.* 2019)	GCA_900492545.1	val
Zea mays	PRJNA697943:<br>SRS8775960<br>SRS8775993<br>SRS8775996	PRJNA412230:<br>SRR6077551<br>SRR6077553<br>SRR6077554	GCF_902167145.1	train

The table contains the species, the BioProject and the linked SRA accessions, including citation if available, of either the ATAC-seq or ChIP-seq experiment passing data preprocessing and quality control, the genome assembly, and the split into training (train), validation (val) or test species. Accessions only used in later development stages are denoted with *.

Table S3: Tissues and treatments.

**Species (scientific)**	**ATAC-seq (tissues/treatments)**	**ChIP-seq (tissues/treatments)**
Actinidia chinensis	PRJNA1039995*:<br>Leaves	PRJNA1039995*:<br>Leaves
Arabidopsis thaliana	PRJNA394532:<br>Roots (Maher *et al.* 2018)<br>PRJNA527732:<br>Leaves (Lu *et al.* 2019)	PRJNA408288:<br>NaN<br>PRJNA535479:<br>Seedlings under cold treatment and following recovery (Xi *et al.* 2020)

Bigelowiella natans	PRJNA753294:<br>Cell culture of unicellular algae<br>(Marinov *et al.* 2022)	
Brachypodium distachyon	PRJNA661629:<br>Leaves under light and dark treatment<br>(An *et al.* 2020)	PRJNA661629:<br>Leaves under light and dark treatment<br>(An *et al.* 2020)
Brassica napus	PRJNA808238:<br>NaN	PRJNA687926:<br>Leaves (Li, Li and Wang 2022)
Brassica oleracea		PRJNA687926:<br>Leaves (Li, Li and Wang 2022)
Brassica rapa		PRJNA687926:<br>Leaves (Li, Li and Wang 2022)
Chlamydomonas reinhardtii		PRJNA681680:<br>NaN
Eragrostis nindensis	PRJNA807505:<br>Desiccated leaves	PRJNA548367:<br>Leaves under drought and sufficient water treatment
Glycine max	PRJNA657378:<br>Leaves (Huang *et al.* 2021)	PRJNA753632:<br>Leaves under normal and salt treatment (Yung *et al.* 2022)
Malus domestica	PRJNA821644:<br>Unknown tissue under drought and sufficient water treatment	PRJNA267727:<br>Field-grown leaves
Marchantia polymorpha	PRJNA597314:<br>Thallus	PRJNA1043823*:<br>Thallus
Medicago truncatula	PRJNA647765:<br>Roots at 0 h, 15 min, 30 min, 1 h, 2 h, 4 h, 8h after *Sinorhizobium meliloti* lipo-chitooligosaccharides treatment (Pereira *et al.* 2022)	PRJNA783892:<br>Whole aerial tissues harvested from 14–17-day-old plants at 4 h after dawn (Jaudal *et al.* 2022)
Oropetium thomaeum	PRJNA807505:<br>Well-watered and desiccated leaves	
Oryza brachyantha		PRJNA521886:<br>Aerial tissue
Oryza sativa	PRJNA751145:<br>Pistil and anther under low and normal temperature treatment	PRJNA386513:<br>Callus, leaves, and panicle
Panicum miliaceum	PRJNA1063172*:<br>Leaves	
Prunus persica		PRJNA381300:<br>Leaf and ripe fruit<br>PRJNA589110:<br>Vegetative bud
Pyrus x bretschneideri		PRJNA669907:<br>Buds during dormancy transition
Sesamum indicum		PRJNA577518:<br>Unknown tissue under light and dark treatment
Setaria italica		PRJNA391551:<br>Leaf mesophyll<br>PRJNA486213:<br>Bundle sheath
Solanum lycopersicum	PRJNA850391:<br>4-weeks-old fourth leaves after 1 h of heat stress (Huang *et al.* 2023)<br>PRJNA937410:<br>Fruit	PRJNA624889:<br>Pericarp from the equatorial part of the fruit
Sorghum bicolor	PRJNA1063172*:<br>Leaves	
Spirodela polyrhiza	PRJNA527732:<br>Leaves (Lu *et al.* 2019)	PRJNA527732:<br>Leaves (Lu *et al.* 2019)
Zea mays	PRJNA697943:<br>Unknown tissue (single cell)	PRJNA412230:<br>Root tips

The tissues and/or treatments used for a given species is listed per NGS dataset and study, including BioProject accession. Entirely unknown tissue and treatment is denoted with NaN. Accessions only used in later development stages are denoted with *.

S3.2 Training parameters

Table S4: Model training parameters.

**Parameters**	**Model**									
	U-Net	Hybrid	Bi-Hybrid	Bi-Hybrid_02	Bi-Hybrid_03.1	Bi-Hybrid_03.2	Bi-Hybrid_04	Bi-Hybrid_05	Com-bined	Com-bined_02
Predmoter commit	cbc2256	cbc2256	cbc2256	cbc2256	cbc2256	cbc2256	c9ee6d7	c9ee6d7	c9ee6d7	137f06a
**Configuration parameters**										
datasets	atacseq	atacseq	atacseq	atacseq	atacseq	atacseq	atacseq	h3k4me3	atacseq, h3k4me3	atacseq, h3k4me3
ram-efficient	false	false	false	false	false	false	false	false	false	false
**Model parameters**										
model-type	cnn	hybrid	bi-hybrid	bi-hybrid	bi-hybrid	bi-hybrid	bi-hybrid	bi-hybrid	bi-hybrid	bi-hybrid
cnn-layers	3	3	3	3	3	3	3	3	3	3
filter-size	64	64	64	64	64	64	64	64	64	64
kernel-size	18	18	18	18	18	18	18	18	18	18
step	3	3	3	3	3	3	3	3	3	3
up	2	2	2	2	2	2	2	2	2	2
dilation	1	1	1	1	1	1	1	1	1	1
lstm-layers	/	2	2	2	2	2	2	2	2	2
hidden-size	/	128	128	128	128	128	128	128	128	128
bnorm	false	false	false	true	true	true	true	true	true	true
dropout	/	0	0	0	0.3	0.5	0.3	0.3	0.3	0.3
learning-rate	0.001	0.001	0.001	0.001	0.001	0.001	0.001	0.001	0.001	0.001
**Trainer/ callback parameters**										
ckpt-quantity	avg_val_accuracy	avg_val_accuracy	avg_val_accuracy	avg_val_accuracy	avg_val_accuracy	avg_val_accuracy	avg_val_accuracy	avg_val_accuracy	avg_val_accuracy	avg_val_accuracy
save-top-k	-1	-1	-1	-1	-1	-1	-1	-1	-1	-1
stop-quantity	avg_train_loss	avg_train_loss	avg_train_loss	avg_train_loss	avg_train_loss	avg_train_loss	avg_train_loss	avg_train_loss	avg_train_loss	avg_train_loss
patience	10	10	10	10	10	10	10	10	10	10
batch size	200	200	200	200	200	200	200	200	200	200
device	gpu	gpu	gpu	gpu	gpu	gpu	gpu	gpu	gpu	gpu
num-devices	2	2	2	2	2	2	2	2	2	2
num-workers	4	4	4	4	4	4	4	4	4	0

The parameter names are the exact naming convention used in Predmoter. The GitHub commit used is listed as Predmoter commit. A detailed explanation of the parameters can be found at: https://github.com/weberlab-hhu/Predmoter/blob/main/docs/Predmoter_options.md.

S3.3 Tabular results

Table S5: List of the best models.

**Model**	**Epoch**	**Seed**	**Validation Pearson's r**
U-Net	6	4227086911	0.4125
Hybrid	147	961333724	0.4370
BiHybrid	104	132709648	0.4884
BiHybrid_02	36	132709648	0.5217
BiHybrid_03.1	61	961333724	0.5336
BiHybrid_03.2	53	961333724	0.5374

BiHybrid_04	49	4227086911	0.5323
BiHybrid_05	2	132709648	0.4387
Combined	20	4227086911	0.4835
Combined_02	22	961333724	0.4927
IS_10	68	961333724	0.5852
IS_20	109	961333724	0.5851
L1O *A. chinensis*	50	961333724	0.4251
L1O *A. thaliana*	56	961333724	0.7083
L1O *B. natans*	35	961333724	0.1247
L1O *B. distachyon*	18	961333724	0.6674
L1O *B. napus*	36	961333724	0.5271
L1O *B.oleracea*	47	961333724	0.5789
L1O *B. rapa*	56	961333724	0.6509
L1O *C. reinhardtii*	67	961333724	-0.0379
L1O *E. nindensis*	31	961333724	0.3956
L1O *G. max*	38	961333724	0.5041
L1O *M. domestica*	38	961333724	0.4182
L1O *M. polymorpha*	24	961333724	0.4474
L1O *M. truncatula*	19	961333724	0.4717
L1O *O. thomaeum*	59	961333724	0.5818
L1O *O. brachyantha*	6	961333724	0.7862
L1O *O. sativa*	40	961333724	0.6019
L1O *P. miliaceum*	88	961333724	0.4352
L1O *P. persica*	25	961333724	0.6724
L1O *P. x bretschneideri*	3	961333724	0.5905
L1O *S. indicum*	21	961333724	0.7435
L1O *S. italica*	48	961333724	0.6289
L1O *S. lycopersicum*	32	961333724	0.3756
L1O *S. bicolor*	29	961333724	0.5658
L1O *S. polyrhiza*	13	961333724	0.5652
L1O *Z.mays*	57	961333724	0.2717

The best model was determined by the highest average validation Pearson correlation coefficient over all three replicates and all epochs (rounded to four decimal points). The epoch numbering uses Python convention, starting at zero. The listed models were used for testing and inference. The model checkpoint files of all models except the leave-one-out cross validation models (L1O) can be found at: https://github.com/weberlab-hhu/predmoter_models.

Table S6: Pearson's correlation for ATAC-seq predictions per species.

**Species (scientific)**	**Model**													**Split**
	U-Net	Hybrid	Bi-Hybrid	Bi-Hybrid_02	Bi-Hybrid_03.1	Bi-Hybrid_03.2	Bi-Hybrid_03.1*	Bi-Hybrid_04*	Com-bined*	Com-bined_02*	L1O*	IS_10*	IS_20*	
Actinidia chinensis	NaN	NaN	NaN	NaN	NaN	NaN	NaN	NaN	NaN	0.4608	0.3908	0.4771	0.4674	train
Arabidopsis thaliana	0.2247	0.3338	0.4797	0.5916	0.6043	0.5881	0.6049	0.6122	0.6106	0.634	0.656	0.695	0.6833	test
Bigelo-wiella natans	0.1043	0.3947	0.4876	0.5875	0.6182	0.5852	0.6182	0.6191	0.5608	0.5571	0.1247	0.556	0.5656	train
Brachy-podium distachyon	0.5337	0.6203	0.6843	0.7265	0.7466	0.7371	0.7469	0.7404	0.7165	0.7545	0.6138	0.7551	0.7577	train
Brassica napus	0.2264	0.2778	0.3904	0.4403	0.4485	0.4371	0.4817	0.4855	0.4777	0.4913	0.4444	0.5342	0.4862	train
Eragrostis nindensis	0.3114	0.4084	0.465	0.5017	0.5323	0.5142	0.5323	0.5335	0.493	0.5009	0.3436	0.4932	0.4862	train
Glycine max	0.5407	0.6222	0.6721	0.7124	0.72	0.7138	0.723	0.7245	0.7174	0.7259	0.5717	0.7298	0.7297	train
Malus domestica	0.2332	0.3524	0.4358	0.4829	0.519	0.4917	0.5185	0.5262	0.4801	0.4885	0.4069	0.4668	0.4695	train
Marchantia polymorpha	0.3984	0.4671	0.5579	0.6037	0.6302	0.6121	0.6302	0.6334	0.5965	0.6174	0.4276	0.6421	0.605	train
Medicago truncatula	0.4268	0.4498	0.4918	0.5363	0.5498	0.5497	0.5504	0.5489	0.5583	0.5657	0.5548	0.6749	0.6598	val
Oropetium thomaeum	0.4873	0.6482	0.7423	0.7713	0.7993	0.7816	0.7993	0.8022	0.76	0.7677	0.5818	0.7326	0.7329	train
Oryza sativa	0.3743	0.46	0.4818	0.5063	0.4926	0.4887	0.493	0.4903	0.4472	0.5853	0.5801	0.6307	0.6693	test
Panicum miliaceum	NaN	NaN	NaN	NaN	NaN	NaN	NaN	NaN	NaN	0.5304	0.4352	0.516	0.4957	train
Solanum lycoper-sicum	0.2897	0.3671	0.4361	0.4832	0.5037	0.4913	0.5077	0.5131	0.4964	0.5042	0.383	0.4928	0.4982	train
Sorghum bicolor	NaN	NaN	NaN	NaN	NaN	NaN	NaN	NaN	NaN	0.6693	0.5658	0.6609	0.6619	train
Spirodela polyrhiza	0.3684	0.397	0.4779	0.4766	0.4836	0.4994	0.4836	0.4813	0.4938	0.5184	0.5367	0.6912	0.707	val
Zea mays	0.3185	0.4383	0.4854	0.5334	0.5469	0.5274	0.5496	0.5504	0.54	0.5519	0.394	0.5571	0.5507	train

The predictions of the best models were compared with the experimental data per species. The intra-species prediction models, IS_10 and IS_20, were evaluated on 10 and 20 % of the data from each species respectively. Only the test metrics for each of the validation species of the 25 leave-one-out cross validation models (L1O) are listed. The resulting Pearson correlation

coefficients were rounded to four decimal points. Gap subsequences were excluded from all test runs. Results marked with * also excluded flagged subsequences.

Table S7: Pearson's correlation for ChIP-seq predictions per species.

Species (scientific)	Model						Split
	Bi-Hybrid_05*	Combined*	Com-bined_02*	L1O*	IS_10*	IS_20*	
Actinidia chinensis	NaN	NaN	0.5963	0.4595	0.6096	0.5996	train
Arabidopsis thaliana	0.7692	0.7641	0.7719	0.7607	0.8019	0.7935	test
Brachypodium distachyon	0.7391	0.7871	0.7972	0.7211	0.807	0.8058	train
Brassica napus	0.6001	0.6222	0.6284	0.6098	0.6808	0.63	train
Brassica oleracea	0.5602	0.5893	0.5945	0.5789	0.5811	0.5704	train
Brassica rapa	0.6328	0.6616	0.6679	0.6509	0.7145	0.6205	train
Chlamy-domonas reinhardtii	0.7102	0.7988	0.8002	-0.0379	0.809	0.8064	train
Eragrostis nindensis	0.4914	0.5603	0.5665	0.4476	0.5712	0.5676	train
Glycine max	0.5097	0.5662	0.5756	0.4365	0.5808	0.5651	train
Malus domestica	0.4454	0.4919	0.4946	0.4295	0.5067	0.4907	train
Marchantia polymorpha	NaN	NaN	0.6710	0.4672	0.6852	0.6398	train
Medicago truncatula	0.3957	0.3771	0.3833	0.3887	0.5396	0.5622	val
Oryza brachyantha	0.7991	0.8372	0.8371	0.7862	0.8238	0.8452	train
Oryza sativa	0.5918	0.6160	0.6245	0.6237	0.6626	0.6862	test
Prunus persica	0.6591	0.7055	0.7110	0.6724	0.7163	0.66	train
Pyrus x bretschneideri	0.6088	0.6328	0.6381	0.5905	0.6029	0.6456	train
Sesamum indicum	0.7589	0.7895	0.7982	0.7435	0.809	0.7843	train
Setaria italica	0.6559	0.7296	0.7451	0.6289	0.727	0.7596	train
Solanum lycopersicum	0.3860	0.4227	0.4273	0.3683	0.4188	0.3925	train
Spirodela polyrhiza	0.5708	0.5699	0.5790	0.5937	0.7304	0.7442	val
Zea mays	0.2873	0.3294	0.3343	0.1494	0.3454	0.3482	train

The predictions of the best models were compared with the experimental data per species. The intra-species prediction models, IS_10 and IS_20, were evaluated on 10 and 20 % of the data from each species respectively. Only the test metrics for each of the validation species of the 25 leave-one-out cross validation models (L1O) are listed. The resulting Pearson correlation coefficients were rounded to four decimal points. Gap subsequences and flagged regions were excluded from all test runs. Results marked with * also excluded flagged subsequences.

S3.4 Peak statistics

Table S8: Peak percentage statistics.

Domain	ATAC-seq peak percentage (training set)	ChIP-seq peak percentage (training set)	
Dicots	5.08	11.24	
Monocots	9	11.03	
Mosses and Algae	8.48	13.32	
Total	5.64	11.62	
Dicots$^+$	5.41	12.77	
Monocots$^+$	7.81	11.03	
Total$^+$	6.99	12.27	
**Species (scientific)**	**ATAC-seq peak percentage**	**ChIP-seq peak percentage**	**Split**

Actinidia chinensis	6.74	25.01	train
Arabidopsis thaliana	16.55	18.42	test
Bigelowiella natans	6.57	/	train
Brachypodium distachyon	12.01	10.89	train
Brassica napus	2.58	14.52	train
Brassica oleracea	/	13.99	train
Brassica rapa	/	15.48	train
Chlamydomonas reinhardtii	/	17.26	train
Eragrostis nindensis	9.4	19.28	train
Glycine max	6.52	8.39	train
Malus domestica	5.24	6.33	train
Marchantia polymorpha	10.39	9.39	train
Medicago truncatula	8.4	5.73	val
Oropetium thomaeum	10.86	/	train
Oryza brachyantha	/	13.43	train
Oryza sativa	7.6	11.08	test
Panicum miliaceum	6.15	/	train
Prunus persica	/	13.16	train
Pyrus x bretschneideri	/	11.93	train
Sesamum indicum	/	13.58	train
Setaria italica	/	9.16	train
Solanum lycopersicum	5.97	4.5	train
Sorghum bicolor	4.68	/	train
Spirodela polyrhiza	14.46	19.04	val
Zea mays	3.74	2.37	train

The base-wise percentages of peaks called from the merged sample bam files per species, domain (training set only) and NGS dataset are listed. Flagged sequences were excluded from the calculations, except for genome assemblies on scaffold or contig level, i.e. *Bigelowiella natans*, *Eragrostis nindensis*, *Marchantia polymorpha*, *Oropetium thomaeum*, *Pyrus x bretschneiderii* and *Spirodela polyrhiza*. Entries denoted with $^+$ include the percentages of additional data added in later development stages, i.e. 3 more ATAC-seq datasets from *Actinidia chinensis*, *Panicum miliaceum* and *Sorghum bicolor*, and 2 more ChIP-seq datasets corresponding to acquired ATAC-seq datasets from *A. chinensis* and *M. polymorpha*.

References

An yunyun, Shen W, Li J *et al.* Dynamic epigenome changes in response to light in Brachypodium distachyon. *Authorea Preprints* 2020, DOI: 10.22541/AU.160336987.75933449/V1.

Collette A. *Python and HDF5*., 2013.

Falcon W. Pytorch Lightning: GitHub. 2019.

Holst F, Bolger A, Günther C *et al.* Helixer–de novo Prediction of Primary Eukaryotic Gene Models Combining Deep Learning and a Hidden Markov Model. *bioRxiv* 2023, DOI: 10.1101/2023.02.06.527280.

Huang M, Zhang L, Zhou L *et al.* An expedient survey and characterization of the soybean JAGGED 1 (GmJAG1) transcription factor binding preference in the soybean genome by modified ChIPmentation on soybean protoplasts. *Genomics* 2021;**113**:344–55.

Huang Y, An J, Sircar S *et al.* HSFA1a modulates plant heat stress responses and alters the 3D chromatin organization of enhancer-promoter interactions. *Nature Communications* 2023;**14**:1–15.

Hunter JD. Matplotlib: A 2D graphics environment. *Computing in Science and Engineering* 2007;**9**:90–5.

Jaudal M, Mayo-Smith M, Poulet A *et al.* MtING2 encodes an ING domain PHD finger protein which affects Medicago growth, flowering, global patterns of H3K4me3, and gene expression. *The Plant Journal* 2022;**112**:1029–50.

Kluyver T, Ragan-Kelley B, Perez F *et al.* Jupyter Notebooks – a publishing format for reproducible computational workflows. In: Loizides F, Schmidt B (eds.). *Positioning and Power in Academic Publishing: Players, Agents and Agendas*. IOS Press, 2016, 87–90.

Letunic I, Bork P. Interactive Tree Of Life (iTOL) v5: an online tool for phylogenetic tree display and annotation. *Nucleic Acids Research* 2021;**49**:W293–6.

Li Z, Li M, Wang J. Asymmetric subgenomic chromatin architecture impacts on gene expression in resynthesized and natural allopolyploid Brassica napus. *Communications Biology 2022 5:1* 2022;**5**:1–16.

Lu Z, Marand AP, Ricci WA *et al.* The prevalence, evolution and chromatin signatures of plant regulatory elements. *Nature Plants* 2019;**5**:1250–9.

Maher KA, Bajic M, Kajala K *et al.* Profiling of Accessible Chromatin Regions across Multiple Plant Species and Cell Types Reveals Common Gene Regulatory Principles and New Control Modules. *The Plant Cell* 2018;**30**:15–36.

Marinov GK, Chen X, Wu T *et al.* The chromatin organization of a chlorarachniophyte nucleomorph genome. *Genome Biology* 2022;**23**:1–18.

Paszke A, Gross S, Massa F *et al.* PyTorch: An Imperative Style, High-Performance Deep Learning Library. *Advances in Neural Information Processing Systems* 2019;**32**, DOI: 10.48550/arxiv.1912.01703.

Pereira WJ, Knaack S, Chakraborty S *et al.* Functional and comparative genomics reveals conserved noncoding sequences in the nitrogen-fixing clade. *New Phytologist* 2022;**234**:634–49.

Rossum GV, Drake FL. Python 3 Reference Manual; CreateSpace. *Scotts Valley, CA* 2009:242.

Stiehler F, Steinborn M, Scholz S *et al.* Helixer: cross-species gene annotation of large eukaryotic genomes using deep learning. *Bioinformatics* 2021;**36**:5291–8.

Waskom ML. seaborn: statistical data visualization. *Journal of Open Source Software* 2021;**6**:3021.

Xi Y, Park SR, Kim DH *et al.* Transcriptome and epigenome analyses of vernalization in Arabidopsis thaliana. *The Plant Journal* 2020;**103**:1490–502.

Yung WS, Wang Q, Huang M *et al.* Priming-induced alterations in histone modifications modulate transcriptional responses in soybean under salt stress. *The Plant Journal* 2022;**109**:1575–90.
